# Selective Inhibition of KRAS^G13C^ Reveals an Increased Dependence on Wild-type *RAS* Isoforms in Codon 13 RAS-Mutant Cancers

**DOI:** 10.1158/2159-8290.CD-25-0886

**Published:** 2026-06-22

**Authors:** Kyle J. Seamon, Yongxian Zhuang, Yu Chi Yang, Sujata Chakraborty, James Cregg, Aidan C.A. Tomlinson, Andrea Gould, Ethan Ahler, Benjamin J. Maldonato, Jessica N. Spradlin, Kristof Pota, Caroline Weller, Abby Marquez, Zhican Wang, Elena S. Koltun, John E. Knox, Adrian L. Gill, Jacqueline A.M. Smith, Mallika Singh, Jingjing Jiang, David Wildes, Matthew Holderfield

**Affiliations:** Revolution Medicines, Inc., Redwood City, California.

## Abstract

Covalent KRAS^G12C^ inhibitors have changed the treatment landscape for patients with non–small cell lung cancer and colorectal cancer, but numerous RAS-mutant cancers lack approved targeted therapies. In this study, we describe RMC-8839, an oral RAS(ON) G13C-selective, covalent, tricomplex inhibitor that induced tumor regressions in selected *KRAS*^G13C^-mutant xenograft models. However, one third of *KRAS*^G13C^-mutant human cancer cell lines *in vitro* showed incomplete RAS pathway suppression despite near-complete *KRAS*^G13C^ engagement, suggesting a role for wild-type RAS(ON). We find that codon 13–mutant *RAS* differs from other *KRAS* mutations, exhibiting decreased stability, increased nucleotide exchange, and substantial intrinsic and guanosine triphosphatase-activating protein–stimulated GTP hydrolysis, which decreases oncogenicity. Furthermore, co-occurring RAS pathway mutations leading to increased wild-type RAS activation are enriched in codon 13–mutant tumors. Consistent with a role for wild-type RAS(ON) signaling, the combination of RMC-8839 with a RAS(ON) multiselective inhibitor resulted in deeper inhibition of *KRAS*^G13C^-mutant xenograft tumor growth than either inhibitor alone.

**Significance:**

An enhanced understanding of the underlying biology of RAS mutations is critical to inform therapeutic strategies. Our preclinical data highlight that codon 13 mutant tumors are more reliant on wild-type RAS signaling than codon 12 tumors and demonstrate that this can be addressed by combining RAS(ON) G13C-selective and multiselective inhibitors.

*See related commentary by Araujo and Skoulidi, p. 2206*

*See related article by McDaid et al., p. 2274*

## Teaser

RMC-8839, an oral, RAS(ON) G13C-selective, covalent, tricomplex inhibitor, exhibits antitumor activity as a single agent and in combination with a RAS(ON) multi-selective inhibitor in *KRAS*^G13C^-mutant preclinical models.

## Introduction

The *RAS* genes (*KRAS*, *NRAS*, and *HRAS*) are among the most commonly mutated proto-oncogenes in human cancers (1). Functioning as master regulators of cell proliferation, RAS proteins are small guanosine triphosphatases (GTPase) that cycle between a GDP-bound (OFF) state and a GTP-bound (ON) state, with the ON state binding to effector proteins to initiate the activation of downstream signaling pathways (2). RAS(OFF) releases GDP and binds GTP through two distinct mechanisms: a slow exchange involving spontaneous release of GDP and a much more rapid guanine nucleotide exchange factor (GEF)–catalyzed reaction promoted by activated receptor tyrosine kinases (RTK; refs. 3, 4). RAS(ON) hydrolyzes GTP to GDP through distinct paths as well: a slow intrinsic hydrolysis mechanism and a rapid GTPase-activating protein (GAP)–catalyzed reaction (5). Twenty-seven *KRAS* mutations with known oncogenic function have been identified in patient tumor samples (6). The majority of RAS mutations in human cancers are gain-of-function alterations at codons 12, 13, and 61. All oncogenic RAS mutations lead to a decrease in the GAP-catalyzed GTP hydrolysis rate and/or an increase in the nucleotide exchange rate, thereby increasing the steady-state levels of RAS(ON) and pro-proliferative signaling (7). Despite the commonality of RAS hotspot mutations as cancer drivers, there is significant heterogeneity among codon 12 (G12X), codon 13 (G13X), and codon 61 (Q61X) mutations with respect to their biochemical, structural, and cellular properties (8–15). The incidence of each oncogenic RAS allele varies by cancer subtype, and mutations emerge under distinct genetic backgrounds, with divergent comutations (16). Some RAS alleles are also known to activate different signaling pathways, suggesting nonoverlapping function (17). Therefore, *KRAS*-mutant cancer cells may not all respond equally to pharmacologic KRAS inhibition.

Currently, the FDA-approved treatments for *KRAS*-mutant cancers are limited to covalent inhibitors that target the oncogenic cysteine introduced in KRAS^G12C^. These inhibitors preferentially bind KRAS^G12C^(OFF) in a pocket adjacent to switch II (SWII). This class now includes approved drugs [sotorasib (18) and adagrasib (19)] and several agents in late-stage clinical development [e.g., divarasib (20) and olomorasib (21)]. By removing the cysteine-targeting covalent warhead and optimizing noncovalent interactions, the SWII pocket has been exploited to design KRAS^G12D^(OFF) inhibitors [e.g., MRTX1133 (22) and LY3962673 (23)] and pan-KRAS(OFF)-selective inhibitors [e.g., BI-2493/BI-2865 (24), PF-07934040, LY4066434 (25)]. All these OFF-state inhibitors bind preferentially to GDP-bound KRAS and block nucleotide exchange back to the active state, a mechanism that first requires the hydrolysis of GTP to GDP by the KRAS-mutant protein being targeted (26).

To target RAS(ON), we have previously reported tricomplex inhibitors that first bind to the cellular chaperone cyclophilin A (CYPA) to generate a neomorphic protein interface that engages RAS(ON) to form an inhibitory tricomplex (27). The binding of the inhibitor CYPA binary complex to RAS directly blocks signaling by sterically occluding the binding of any effectors (e.g., RAF kinases). The tricomplex platform was first applied to the G12C mutant by introducing a cysteine-targeting warhead to a CYPA-binding scaffold, with further elaboration resulting in the investigational RAS(ON) G12C-selective inhibitor elironrasib (RMC-6291). Optimization of the noncovalent affinity led to the development of the RAS(ON) multiselective inhibitors RMC-7977 (28) and daraxonrasib (RMC-6236; ref. 29) that bind with near-equal affinity to wild-type and common oncogenic mutants of RAS. Unlike the SWII pocket inhibitors, the tricomplex binding site is not divergent among KRAS, NRAS, and HRAS, so tricomplex RAS(ON) inhibitors have activity on all three RAS isoforms. The RAS(ON) multiselective inhibitor daraxonrasib, the RAS(ON) G12C-selective covalent inhibitor elironrasib, and the RAS(ON) G12D-selective covalent inhibitor zoldonrasib (RMC-9805) are investigational agents currently under clinical evaluation.

To extend the RAS(ON) tricomplex platform to additional RAS-mutant proteins, we looked to codon 13 mutations, a subset that is somewhat less sensitive than codon 12 mutants to the RAS(ON) multiselective inhibitors in preclinical studies (28, 29). We targeted *KRAS*^G13C^, a mutation associated with non–small cell lung cancers (NSCLC; ref. 30), as it introduces a cysteine residue that can be readily targeted covalently. Building on experience generating RAS(ON) G12C-selective inhibitors (27), we used structure-guided drug design to discover RMC-8839, a RAS(ON) G13C-selective covalent inhibitor. Using RMC-8839 in conjunction with structural and biochemical studies, we find that codon 13 RAS mutations comprise a class with decreased protein stability, increased spontaneous nucleotide exchange, and substantial GTP hydrolysis activity. Collectively, these properties result in decreased tumorigenic potential for G13X-mutant RAS proteins that is compensated for by a concomitant increase in wild-type RAS signaling, as evidenced by an increase in co-occurring neurofibromin 1 (*NF1*) loss-of-function or *BRAF* gain-of-function mutations in patients with codon 13 RAS-mutant tumors. The treatment of a panel of *KRAS*^G13C^-mutant cancer cell lines with RMC-8839 revealed a highly heterogeneous effect on cell signaling, consistent with the cooperative activity of mutant and wild-type RAS signaling being a shared characteristic of codon 13 tumors. Finally, using this biological understanding, we demonstrate that combining RMC-8839 to maximally inhibit the KRAS^G13C^-mutant protein and RMC-7977 or daraxonrasib to inhibit wild-type RAS results in deeper and more durable suppression of KRAS^G13C^ tumor xenograft signaling and growth compared with either agent alone.

## Results

### Biochemical Basis of Cellular Transformation by RAS Mutations

Previous reports have indicated that not all RAS oncoproteins are functionally interchangeable, with biochemical and structural differences both among substitutions at different codons and among different substitutions at the same codon (8). As a first step toward covalently targeting KRAS^G13C^, we solved structures of 5′-guanylyl imidodiphosphate (GMPPNP)–bound wild-type and G13C-mutant KRAS(ON) to mimic the active, GTP-bound state (Fig. 1A; Supplementary Table S1). Despite strong structural similarity between wild-type and mutant KRAS (RMSD ∼1.5 Å), there are differences in the local conformation around the mutated residue. The ϕ and ψ dihedral angles of codon 12 are in a favorable orientation for glycine or any non-proline amino acid for both wild-type and G13C-mutant KRAS structures. In contrast, the wild-type structures (two proteins in the asymmetric unit) adopt dihedral angles that are only compatible with a glycine at codon 13 (Fig. 1B). For KRAS^G13C^ and consistent with what has been shown previously for G13D mutations (13), the dihedral angles for codon 13 change to accommodate the steric bulk of a side chain (Fig. 1C), suggesting conformational strain that could introduce unique biochemical properties to KRAS^G13X^ proteins.

**Figure 1.**
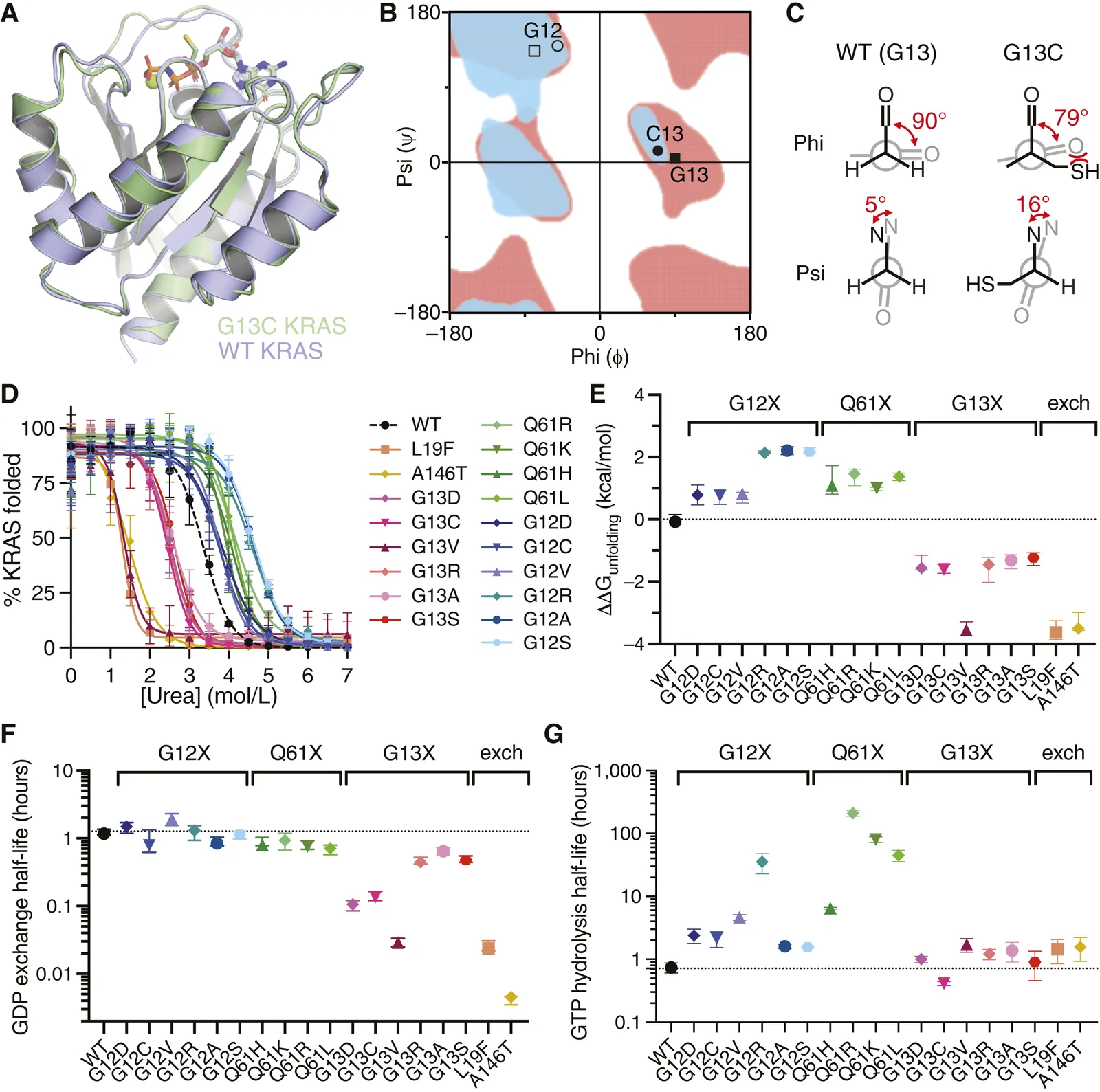
Structural and biochemical properties of mutant KRAS proteins. **A,** Overlaid crystal structures of wild-type (WT) KRAS (green) and KRAS^G13C^ (light blue). **B,** Ramachandran analysis of the dihedral angles of the codon 12 residues (open symbols) and the codon 13 residues (filled symbols) from the crystal structures of WT (circle) and G13C-mutant (square) crystal structures. The energetically favorable regions for generic amino acids (shaded blue) and glycine (shaded pink) are shown (31). **C,** Depiction of the phi and psi angles for codon 13 by Newman projection as obtained from the WT and G13C-mutant crystal structures. **D,** The fraction of KRAS-mutant protein remaining folded as assessed by pulse proteolysis for varying concentrations of urea (representative of *n* = 3). **E,** The ΔΔG_unfolding_ for each mutant relative to WT KRAS calculated from the concentration of urea resulting in half-maximal unfolding of the KRAS-mutant proteins (C_m_) from **D**. **F,** The first-order rate constant for the exchange of mantGDP for the GTP-analog GMPPNP for KRAS-mutant proteins at 37°C. **G,** The first-order rate constant for the hydrolysis of GTP to GDP for KRAS-mutant proteins at 37°C.

To understand the functional consequences of this torsional strain, we systematically characterized the biochemical properties of wild-type KRAS and 18 commonly occurring cancer-associated KRAS variants spanning codons 12, 13, and 61. To identify any impact the oncogenic point mutations may have on protein folding, we used urea denaturation and pulse proteolysis to estimate structural stability (32). All common G12X mutants tested were equally or slightly more stable in their folded state compared with the wild-type proteins (Fig. 1D and E). Similar results were also obtained with all the common Q61X mutations. However, the G13X mutations substantially destabilize the folded state of KRAS, particularly the G13V mutation that introduces a bulky β-branched side chain. Despite this decrease in structural stability in biochemical assays, in studies of KRAS^G12C^ or KRAS^G13C^, we find that both had a similarly long cellular half-life of ∼24 hours (Supplementary Fig. S1A and S1B). We also included L19F and A146T in this panel of KRAS mutants, as these have been observed in patients with RAS mutant cancers and have been reported to exhibit rapid spontaneous nucleotide exchange (33, 34). These “hyperexchange” variants, as well as G13V, were also both severely destabilized in their folded form compared with wild-type RAS (Fig. 1D and E).

The connection between decreased protein stability and increased nucleotide exchange of the known “hyperexchange” variants prompted us to explore whether all G13X mutants have increased spontaneous nucleotide exchange, as has been reported previously for KRAS^G13D^ (13). Indeed, the G13X mutants had higher rates of nucleotide exchange, as did L19F and A146T, whereas all G12X and Q61X mutants had similar GDP to GTP nucleotide exchange rate constants compared with the wild-type protein (Fig. 1F; Supplementary Fig. S2A). In particular, the larger side chains of G13C and G13D had higher exchange rate constants, with the bulky β-branched residue G13V showing the most substantial impact among the codon 13 variants. The correlation between increased nucleotide exchange and decreased protein stability (Supplementary Fig. S2B) suggests that high rates of spontaneous protein unfolding and refolding, akin to the function of GEFs, prompt the release of bound GDP and the binding of the more abundant GTP.

We next turned to measuring the intrinsic GTP hydrolysis rate constants. Consistent with previous reports (8, 33), each of the G13X and the “hyperexchange” mutant KRAS proteins demonstrated similar intrinsic hydrolysis rate constants to wild-type proteins, whereas all G12X mutations were impaired to varying degrees. The smallest substitutions, G12A and G12S, had a modest effect, whereas G12R showed the slowest GTP hydrolysis rate constant among the codon 12 substitutions (Fig. 1G; Supplementary Fig. S2C). The Q61X mutants, expected to be the most disruptive due to the known role of Q61 in the hydrolysis mechanism (35), showed an unexpected diversity in intrinsic hydrolysis rate constants. Specifically, although Q61L, Q61R, and Q61K had exceptionally low rate constants (on the order of G12R), Q61H had a hydrolysis rate constant similar to that of G12V. Collectively, these data demonstrate that the intrinsic biochemical properties of the G12X, G13X, and Q61X classes of RAS mutations are unique and nonoverlapping, and even within each codon, there is variation that likely explains, in part, the prevalence and diverse features of RAS mutant tumors (14, 16).

### Codon 13 RAS-Mutant Proteins Are More Responsive to GAPs

KRAS^G13D^ and KRAS^G13C^ were previously reported to be more susceptible than other KRAS oncoproteins to stimulation of GTP hydrolysis by NF1 GAP (12), and we suspected this may be true of G13X mutants as a class. To assess the sensitivity of each mutant RAS protein to GAPs, a fixed amount of GTP-loaded RAS was incubated for 20 minutes in the presence of varying concentrations of the isolated GAP-related domain (GRD) of NF1 or p120 GAP (RASA1). The EC_50_ (concentration of each GAP protein needed to produce a 50% reduction in GTP-loaded RAS) was used to compare sensitivity. Several G13X proteins had a detectable stimulation by RASA1, with G13A and G13S being 10× to 20× and G13C being 120× less sensitive than wild type. By comparison, the EC_50_s of G12X and Q61X proteins were greater than 10 μmol/L, indicating a >400-fold loss compared with wild-type KRAS of RASA1 GAP sensitivity (Fig. 2A). The G13X mutants showed the greatest sensitivity to stimulation by NF1 GAP, ranging from only 10× to 40× less responsive than wild-type KRAS protein, except for G13R, which was relatively unresponsive. The G12X mutants were less sensitive and exhibited a range of responses, with G12S and G12A being the most sensitive, followed by G12C, then G12D, and finally G12V (200-, 260-, 640-, 950-, and 4,000-fold, respectively). NF1-stimulated GTP hydrolysis by G12R was undetectable (Fig. 2B). The Q61X mutants had no detectable response to NF1-stimulated hydrolysis, except Q61H, which was 1,000-fold less sensitive than wild-type KRAS. These trends largely mirror the intrinsic hydrolysis data (Fig. 1G) and suggest that at least for some oncogenic RAS mutants, GAP-stimulated hydrolysis may modulate intracellular RAS(ON) levels. To test this, we used a nanoBRET-based reporter of the RAS-RAF1 [RAS-binding domain (RBD)] interaction to indirectly measure the levels of RAS(ON) within a cell. RAS mutant plasmids were cotransfected with varying levels of plasmid encoding the GTPase activating domain (GRD) of NF1, and the relative level of RAS-RAF interaction was measured. Consistent with the biochemical data, we found that wild-type KRAS was the most sensitive to NF1 GRD-stimulated hydrolysis, followed by KRAS^G13C^ and KRAS^G13D^, with comparatively little effect observed for KRAS^G12D^, KRAS^G12C^, and KRAS^Q61H^ (Fig. 2C). This effect was specific to the hydrolysis-stimulating activity of NF1, as the same experiment with a catalytically inactive mutant NF1 GRD showed no effect on RAS-RAF levels for any KRAS mutant (Supplementary Fig. S3).

**Figure 2.**
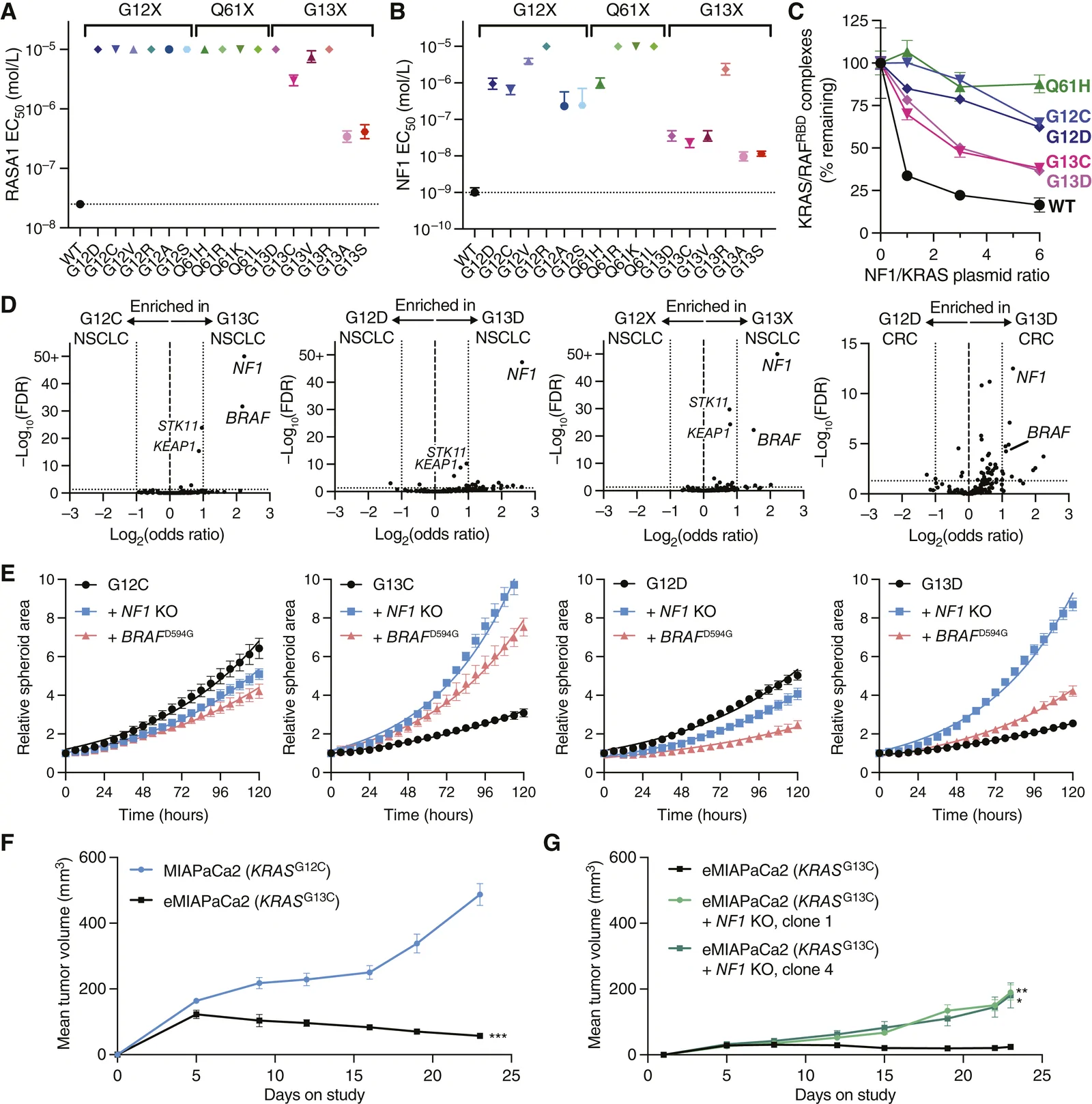
KRAS^G13X^ has reduced oncopotency. The concentration of GAP required to convert half of the RAS(ON) to RAS(OFF) (EC_50_) is plotted for NF1 (**A**) and RASA1 (**B**) across wild-type (WT) and mutant KRAS proteins. The highest concentration tested was 10^−5^ mol/L, so it is the upper limit. **C,** Cellular levels of RAS(ON) assessed through a RAS-RAF nanoBRET signal upon cotransfection of varying ratios of NF1 GAP domain and KRAS plasmids. **D,** Comutations in patients with *KRAS*^G12C^ NSCLC vs. *KRAS*^G13C^ NSCLC, *KRAS*^G12D^ vs. *KRAS*^G13D^ NSCLC, *KRAS*^G12X^ vs. *KRAS*^G13X^ NSCLC, or *KRAS*^G12D^ vs. *KRAS*^G13D^ colorectal cancer compared by log_2_(odds ratio) and FDR from a *χ*^2^ test followed by the Benjamini–Hochberg procedure. Lines indicate log_2_(odds ratio) < −1 or >1 and *P* value < 0.05 (see “Methods”). **E,** Parental or engineered MIA PaCa-2 cells containing the indicated *KRAS* mutation in combination with *NF1* knockout or BRAF^D594G^ expression were monitored for growth in spheroid culture. **F,** Parental MIA PaCa-2 (*KRAS*^G12C^) or engineered *KRAS*^G13C^-mutant MIA PaCa-2 cells were implanted subcutaneously in NOD-SCID mice, and tumor size was assessed (*n* = 5 per group). **G,** Engineered *KRAS*^G13C^-mutant MIA PaCa-2 cells and two clonal cell lines with NF1 knockout were implanted subcutaneously in NOD-SCID mice, and tumor size was measured (*n* = 6 per group). **F-G** *, *P* < 0.05; **, *P* < 0.01; ***, *P* < 0.001 two way repeated measures ANOVA compared to the control on the last treatment day of the control group.

### Comutation Patterns for G12X- and G13X-Mutant Tumors

To further understand differences in the biology of G12X- and G13X-mutant tumors, we looked at the comutation profiles from *RAS*-mutant tumors with either *KRAS*^G12X^ or *KRAS*^G13X^. The glycine to cysteine mutations G12C and G13C often result from a smoking-induced gene signature and occur predominantly in NSCLC, with a lower frequency in colorectal cancer (16). The glycine to aspartate mutations G12D and G13D have the highest incidence in colorectal cancer and are less frequently observed in NSCLC, with G12D also being frequently observed in pancreatic ductal adenocarcinoma (36). Given that the number and nature of cancer-associated mutations are highly tumor type–dependent (16), we compared comutations across KRAS-mutant genotypes within the same histotype. Comparison of co-occurring mutations with a known or likely functional effect (see “Methods”) in *KRAS*^G13C^- and *KRAS*^G12C^-mutant NSCLC revealed a significantly increased comutation frequency of *NF1* or *BRAF* with G13C (Fig. 2D). Virtually all the *BRAF* mutations observed were class II/III, not the more strongly activating class I (Supplementary Table S2; ref. 37). When the analysis was repeated with *KRAS*^G13D^- versus *KRAS*^G12D^-mutant NSCLC, a similarly strong enrichment of NF1 comutations with G13D was observed. Finally, a comparison of all *KRAS*^G13X^ versus *KRAS*^G12X^ genotypes in NSCLC again revealed a strong enrichment of *NF1* comutations in G13X tumors, with a weaker but significant enrichment of *BRAF* comutations. Although *STK11* and *KEAP1* did not meet the cutoff of a 2-fold change in odds ratio, there is a significant increase in mutations in these genes with codon 13 KRAS-mutant NSCLC in these data, as has been reported in a previous analysis (38). In colorectal cancer, comparing G13D to G12D, we again found the strongest enrichment between G13D and *NF1* comutations although there are several additional genes that also met the significance criteria, including *BRAF* mutations, which were predominantly class II/III (Fig. 2D; Supplementary Table S3). Mutations in RTKs, which also activate the MAPK pathway, were slightly enriched in G13X versus G12X NSCLC and G13D versus G12D colorectal cancer, consistent with previous reports (Supplementary Table S4; ref. 12). The identification of a significant co-occurrence of *KRAS*^G13X^ mutations with other mutations in the MAPK pathway may be indicative of reduced oncopotency of codon 13 mutants and a requirement for additional alterations to fully activate the pathway.

### Codon 13 RAS Mutations Have Reduced Oncopotency

To look at differences between *KRAS*^G12X^ and *KRAS*^G13X^ variants in a cellular context, we engineered an isogenic set of MIA PaCa-2 cells that express the native *KRAS*^G12C^, *KRAS*^G12D^, *KRAS*^G13C^, or *KRAS*^G13D^. The *KRAS*^G12X^ cell lines had faster spheroid growth compared with the *KRAS*^G13X^ equivalent [Fig. 2E (black lines)]. However, disruption of the *NF1* locus or introduction of the class III mutation *BRAF*^D594G^ enhanced cellular growth of the G13X lines. In contrast, cellular growth of the G12X lines was impaired upon *NF1* loss or *BRAF*^*D549G*^ expression [Fig. 2E (blue and pink lines)]. These results are consistent with what was observed in patient data in which G13X but not G12X mutations co-occur with *NF1* and *BRAF* class II/III mutations and suggest that the reduced oncopotency of G13X *RAS* mutations can be augmented by additional mechanisms to activate the MAPK pathway. Conversely, the mutual exclusivity observed for *KRAS*^G12X^ and *NF1* or *BRAF* mutations may lead to overactivated KRAS signaling, which can result in oncogene-induced senescence (39).

To extend these results to an *in vivo* tumor formation context, we implanted either parental *KRAS*^G12C^ or engineered *KRAS*^G13C^ MIA PaCa-2 cells subcutaneously into immunodeficient mice and monitored tumor growth. The parental *KRAS*^G12C^ cells exhibited robust tumor growth, whereas the engineered *KRAS*^G13C^ cell line could not sustain growth *in vivo* within 24 days after implantation (Fig. 2F and G). In contrast, when two clones of the engineered *KRAS*^G13C^ MIA PaCa-2 cell line with *NF1* knockout were implanted under the same conditions, they grew robustly although more slowly than the parental *KRAS*^G12C^ lines (Fig. 2G). Collectively, the clinical genomics, *in vitro*, and *in vivo* experimental data suggest that codon 13 RAS mutations are less potent oncogenes than codon 12 mutations and therefore function in conjunction with other mechanisms to sufficiently activate MAPK signaling to drive tumor growth.

### Discovery of a Potent, Covalent, and Selective Oral RAS(ON) G13C-Selective Inhibitor RMC-8839

An early RAS(ON) G12C-selective tricomplex inhibitor (27) with a cysteine-targeting warhead (Cmpd-1; Fig. 3A) showed sufficient interaction with KRAS^G13C^ bound to the nonhydrolyzable GTP analog GMPPNP to enable determination of a crystal structure. Although Cmpd-1 covalently modifies GMPPNP-bound KRAS^G12C^(ON)(27), no covalent bond formation was observed with KRAS^G13C^(ON) due to poor acrylamide positioning and the steric bulk of Y32 adjacent to the G13C residue (Fig. 3B). Comparing the crystal structures of KRAS^G12C^ and KRAS^G13C^, we observed that the cysteine at codon 13 is farther from the CYPA-binding macrocycle of Cmpd-2, suggesting that an extended linker between the macrocycle and warhead might enable efficient modification of KRAS^G13C^. The linker must also be directed to cysteine 13 while avoiding a steric clash with the Y32 residue (Fig. 3B). To accomplish this, the pyrrolidine linker of Cmpd-1 was replaced with the spirocyclic linker of Cmpd-2. The linker replacement substantially enhanced the rate of cross-linking KRAS^G13C^(ON) (Fig. 3C), which could be rationalized by the decreased conformational flexibility of the acrylamide warhead and the optimal positioning of this rigid linker around the Y32 residue in the KRAS^G13C^:Cmpd-3:CYPA crystal structure (Fig. 3D). Further optimization of the cross-linking kinetics, along with drug-like properties, resulted in RMC-8839 (Fig. 3E). A crystal structure of the tricomplex with RMC-8839, CYPA, and KRAS^G13C^(ON) demonstrated that this molecule covalently modifies *KRAS*^G13C^ while retaining the same protein–protein contacts between RAS and CYPA that were seen previously with elironrasib (Supplementary Fig. S4; ref. 27).

**Figure 3.**
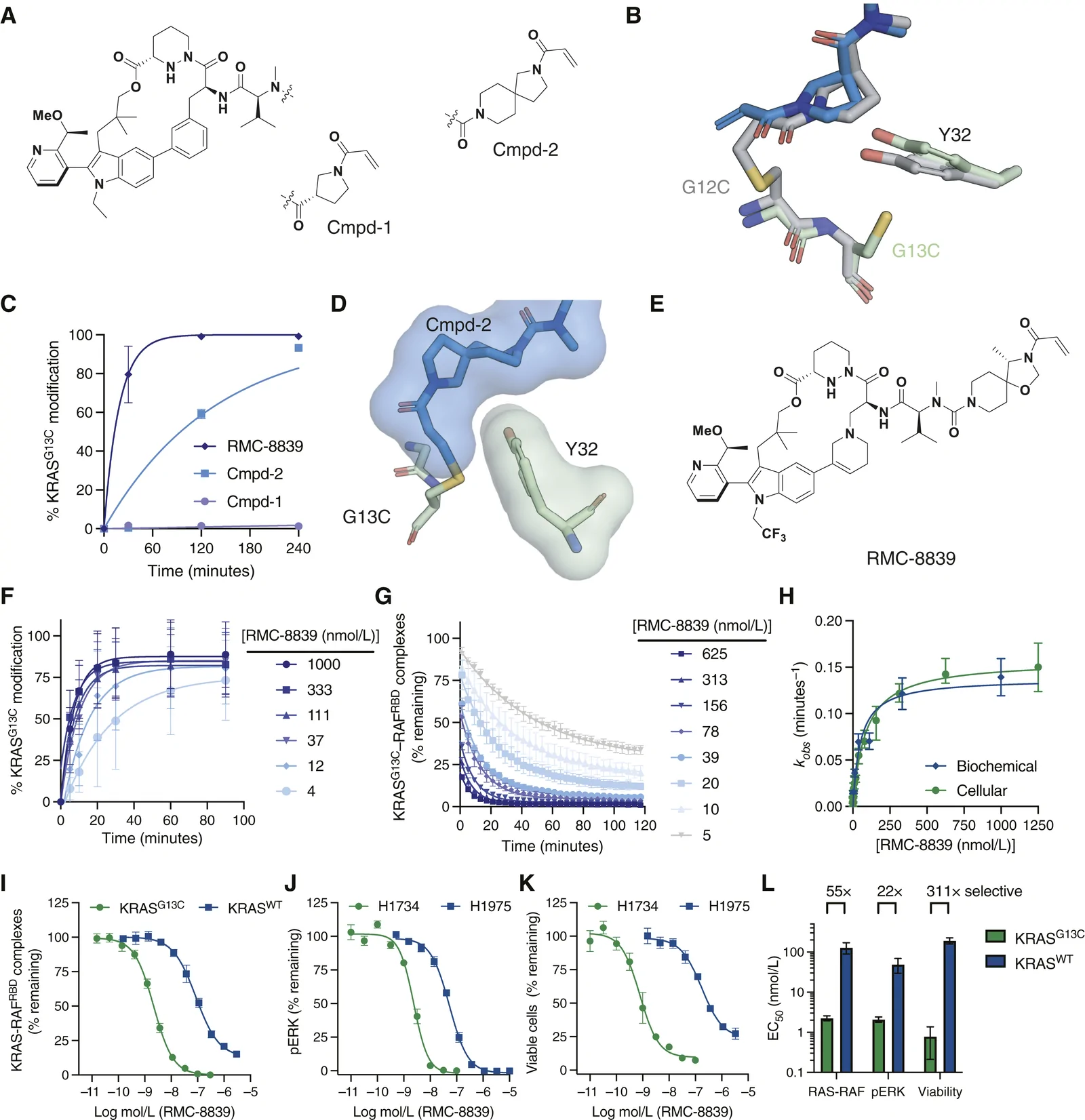
Development of the covalent RAS(ON) G13C-selective inhibitor RMC-8839. **A,** Chemical structures of Cmpd-1 as previously published (27) and Cmpd-3, designed to target KRAS^G13C^. **B,** Overlay of CYPA–compound–KRAS tricomplex crystal structures shows covalent bond formation between Cmpd-1 and KRAS^G12C^ but not between Cmpd-1 and KRAS^G13C^. **C,** Time-dependent covalent modification of KRAS^G13C^ (1 μmol/L) in the presence of compounds (10 μmol/L) and CYPA (25 μmol/L) as assessed by mass spectrometry. **D,** Cmpd-2 tricomplex crystal structure reveals that the spirocyclic linker of Cmpd-2 achieves efficient covalent modification by projecting around KRAS^Y32^ to place the acrylamide warhead near G13C. **E,** Chemical structure of RMC-8839. **F,** Time-dependent covalent modification of KRAS^G13C^ (0.5 nmol/L) in the presence of varying concentrations of RMC-8839 and excess CYPA (25 μmol/L) as assessed by gel electrophoresis mobility shift. **G,** The kinetics of cellular KRAS^G13C^–RAF(RBD) complex disruption were assessed by nanoBRET at varying concentrations of RMC-8839. **H,** The observed rate of biochemical KRAS^G13C^ covalent modification or cellular KRAS^G13C^–RAF(RBD) complex disruption is depicted for varying concentrations of RMC-8839. The selectivity of RMC-8839 was assessed using (**I**) KRAS^G13C^-RAF(RBD) or KRAS^WT^-RAF(RBD) cellular nanoBRET assay after 4 hours of treatment. **J,** NCI-H1734 or NCI-H1975 intracellular pERK to total ERK levels after 4 hours of treatment and (**K**) NCI-H1734 or NCI-H1975 cellular proliferation assays after 5 days of treatment. **L,** The potency (EC_50_) of RMC-8839 for KRAS^G13C^-mutant or KRAS^WT^ across the cellular RAS-RAF nanoBRET, pERK, and proliferation assays shown in **I–K,** respectively. WT, wild type.

To characterize the kinetic parameters of covalent modification of KRAS^G13C^(ON) by RMC-8839 *in vitro*, biochemical reactions were carried out in the presence of excess recombinant CYPA with varying concentrations of RMC-8839 (Fig. 3F). Next, we used a nanoBRET assay to track the kinetics of the disruption of the interaction between KRAS^G13C^ and RAF1 (RBD) in cells after the addition of RMC-8839 (Fig. 3G). The rates and concentration dependence of biochemical covalent modification and cellular KRAS^G13C^-RAF1 RBD disruption kinetics were similar (Fig. 3H). The biochemical *k*_*inact*_ of RMC-8839 for KRAS^G13C^(ON) is 2.83 ± 0.46 × 10^−3^ s^−1^, and the *K*_*I*_ is 28.3 ± 19.7 nmol/L, yielding a *k*_*inact*_/*K*_*I*_ of roughly 10^5^ (mol/L)^−1^s^−1^. RMC-8839 inhibited KRAS^G13C^ more potently than wild-type RAS in a cellular RAS-RAF1 RBD disruption assay (Fig. 3I) and more potently inhibited phospho-ERK (pERK; Fig. 3J) and cell proliferation (Fig. 3K) in a cell line harboring a KRAS^G13C^ mutation (NCI-H1734) compared with an EGFR-mutant, wild-type RAS cell line (NCI-H1975). RMC-8839 was 22- to 311-fold selective for KRAS^G13C^ over wild-type RAS, depending on the assay (Fig. 3L). A panel of KRAS-mutant NSCLC cell lines demonstrated that RMC-8839 is only ∼6× selective for G13C over G12C, as this mutation retains a cysteine residue that can be covalently targeted but is 20× to >100× selective for G13C over all other KRAS mutants tested (Supplementary Fig. S5). RMC-8839 was also more than 1,000-fold more potent in inhibiting the proliferation of NCI-H1734 cells compared with BRAF^V600E^-mutant, RAS-independent A375 cells (Supplementary Fig. S6).

### RMC-8839 Has Antitumor Activity in KRAS^G13C^-Mutant Xenografts

To assess the activity of RMC-8839 *in vivo*, mice bearing NCI-H1734 (*KRAS*^G13C^, NSCLC) xenograft tumors were administered a single oral dose of RMC-8839 ranging from 10 to 100 mg/kg. Dose-dependent increases in plasma RMC-8839 exposure were observed (Fig. 4A). At 100 mg/kg, RMC-8839 demonstrated rapid and durable activity, achieving tumor target engagement (TE) of more than 90% within 3 hours that was maintained above 75% for 24 hours (Fig. 4B). Consistent with TE, tumor RAS-MAPK pathway suppression (as assessed by human *DUSP6* mRNA levels) reached >95% and was maintained above 80% for 24 hours. To evaluate effects on tumor growth, mice were administered RMC-8839 orally once daily for 28 days. RMC-8839 demonstrated a dose-dependent increase in tumor growth inhibition, with a 100 mg/kg daily dose leading to tumor stasis (Fig. 4C). All doses of RMC-8839 were well tolerated, as indicated by stable mouse body weight and lack of clinical signs (Supplementary Fig. S7). RMC-8839 (100 mg/kg every day) also showed antitumor activity in the *KRAS*^G13C^-mutant NSCLC patient-derived xenograft (PDX) model ST2822B, with tumor regressions at 28 days of treatment (Fig. 4D). Pharmacodynamic (PD) analysis of end-of-study tumors from the NCI-H1734 efficacy study (Fig. 4C) was performed using pERK IHC. Tumors collected 8 hours after the final dose of 25 and 100 mg/kg RMC-8839 showed sustained suppression of RAS pathway signaling following 4 weeks of treatment (Supplementary Fig. S8).

**Figure 4.**
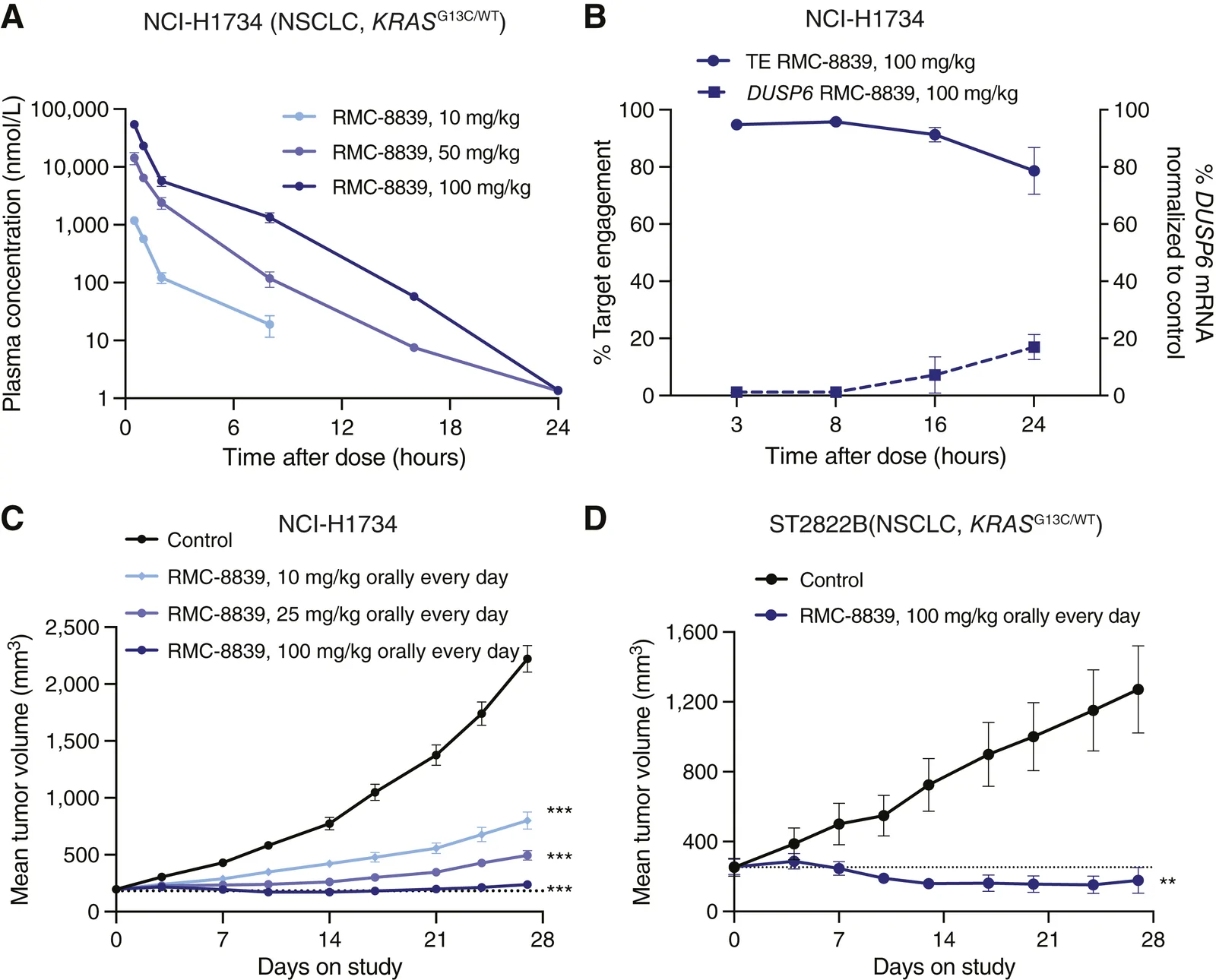
RMC-8839 has activity against *KRAS*^G13C^ tumor xenografts. **A,** Mice bearing NCI-H1734 CDX tumors were treated with a single oral dose of RMC-8839, and the plasma concentrations of RMC-8839 were monitored (mean ± SEM, *n* = 3). **B,** NCI-H1734 tumors excised at various times after a single 100 mg/kg dose of RMC-8839 were subjected to LC/MS to quantify the covalent modification of KRAS^G13C^ and the expression of *DUSP6* mRNA (mean ± SEM, *n* = 3). Mice bearing (**C**) NCI-H1734 CDX tumors (*n* = 10 per group) or (**D**) ST2822B PDX tumors (*n* = 5 per group) were orally administered RMC-8839 daily, and the tumor volume was assessed for 28 days (mean ± SEM). WT, wild type. (**C-D**) **, *P* < 0.01; ***, *P* < 0.001 two-way repeated measures ANOVA compared to the control on the last treatment day of the control group.

### Heterogeneous Signaling Responses to RMC-8839 in *KRAS*^G13C^-Mutant Cell Lines

To examine RMC-8839 activity across a broader set of *KRAS*^G13C^-mutant models, we assessed MAPK signaling in a panel of six cell lines after 4 hours of compound treatment. Most (4/6) of the *KRAS*^G13C^-mutant cell lines showed strong pathway suppression, as evidenced by a reduction in pERK upon covalent modification of the mutant *KRAS* protein (Fig. 5A). The wild-type RAS cell line NCI-H1975 did not respond to RMC-8839 at these concentrations, consistent with the mutant selectivity (Supplementary Fig. S9). Of the *KRAS*^G13C^ cell lines, minimal inhibition of pERK was observed in NCI-H1355 and TOV-21G cells, despite almost complete (85%–95%) covalent modification of KRAS^G13C^, as evidenced by the upward band shift of KRAS in the blots and mass spectrometry (Fig. 5A; Supplementary Fig. S10). Strikingly, pERK in NCI-H1355 and TOV-21G was completely suppressed by the RAS(ON) multiselective inhibitor RMC-7977, suggesting that the remaining ERK phosphorylation after RMC-8839 treatment was wild-type RAS–dependent. However, the published mutation profiles of these cell lines (Supplementary Table S5; ref. 40) do not reveal a clear genomic marker predicting wild-type RAS activation. In addition to these differences in acute pathway suppression and more consistent with the results of previous G12C- or G12D-selective RAS inhibitors (27, 41), some G13C-mutant cells exposed to RMC-8839 for 24 hours exhibited MAPK pathway reactivation at later time points (Supplementary Fig. S11). Despite the variability in acute and longer-term responses to RMC-8839, profiling of phospho-RTKs showed only low-level RTK activation that was not consistently increased upon treatment (Supplementary Figs. S12 and S13).

**Figure 5.**
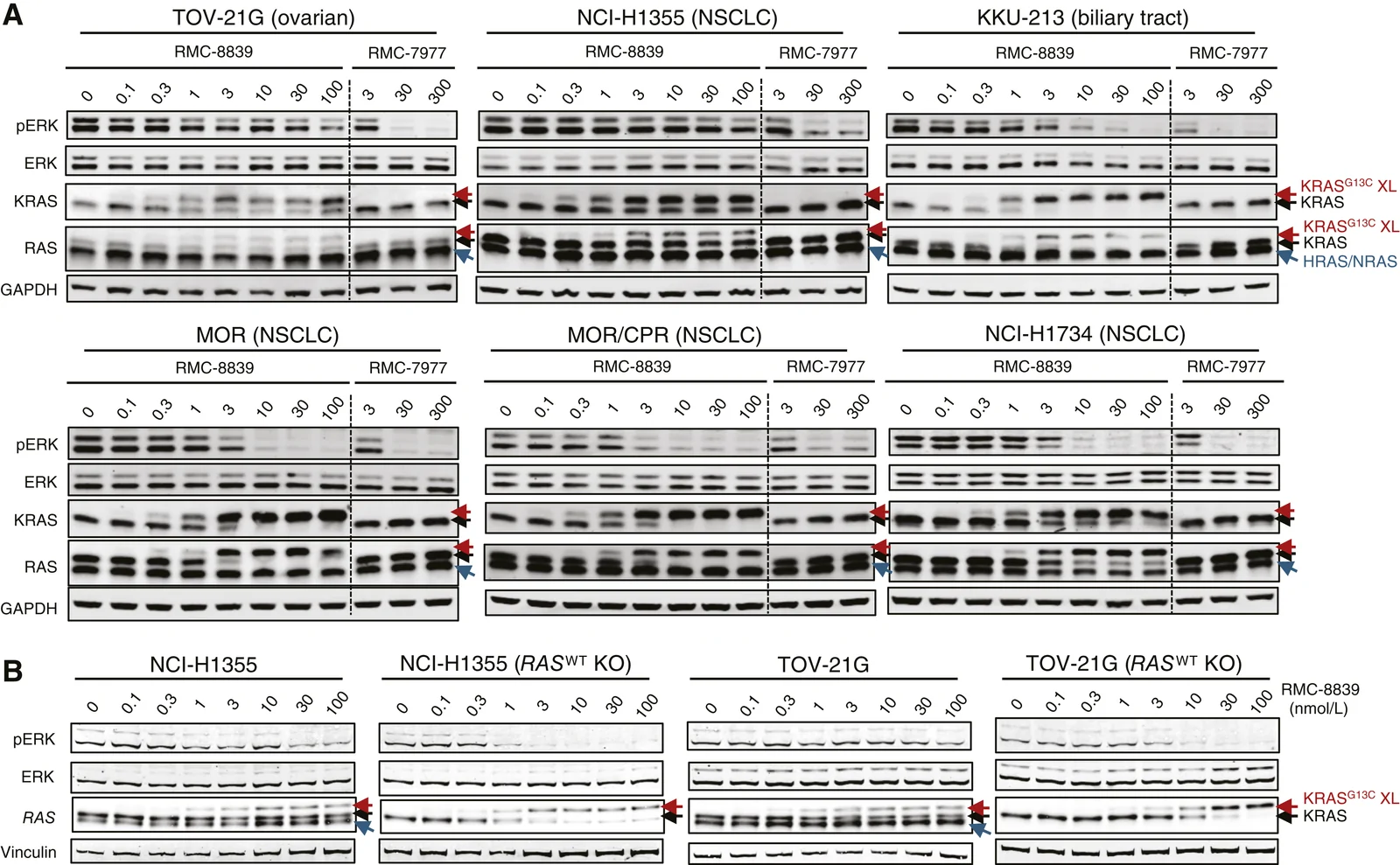
MAPK signaling is driven by mutant and wild-type (WT) RAS isoforms in KRAS^G13C^-mutant cancer cell lines. **A,** KRAS^G13C^-mutant lung adenocarcinoma (NCI-H1734, NCI-H1355, MOR, MOR/CPR), clear-cell ovarian cancer (TOV-21G), or cholangiocarcinoma (KKU-213) cell lines were treated with the indicated concentrations of RMC-8839 or RMC-7977 for 4 hours, then lysed and subjected to immunoblotting with the indicated antibodies. **B,** NCI-H1355 or TOV-21G cell lines were modified with CRISPR to knock out all WT RAS isoforms (NRAS, HRAS, and WT KRAS). These engineered cell lines were treated with RMC-8839 for 4 hours, then lysed and subjected to immunoblotting with the indicated antibodies. The separate bands corresponding to NRAS/HRAS, KRAS, and covalently modified KRAS are indicated on RAS blots.

To gain further insight into RAS signaling, the total and active GTP-loaded RAS levels were measured under basal and inhibitor-treated conditions. NCI-H1734 cells, which are highly dependent on KRAS G13C for sustained pERK signaling, showed very low levels of active wild-type NRAS or HRAS prior to treatment (Supplementary Fig. S14). Conversely, TOV-21G and NCI-H1355 cells, which only partially suppressed pERK after RMC-8839 treatment (Fig. 5), both showed significantly higher levels of active wild-type RAS (Supplementary Fig. S15). After 48 hours of treatment with RMC-8839, all three cell lines showed decreases in active KRAS consistent with targeting the mutant protein although the heterozygous cell lines TOV-21G and NCI-H1355 showed residual active KRAS consistent with the inability of RMC-8839 to target wild-type KRAS. Furthermore, TOV-21G and NCI-H1355 showed a further compensatory activation of wild-type RAS proteins that did not occur in the fully responsive NCI-H1734 cell line (Supplementary Fig. S14).

To confirm the hypothesis that active wild-type RAS proteins were contributing to the residual pERK signaling, we generated NCI-H1355 and TOV-21G cell lines in which wild-type *KRAS*, *NRAS*, and *HRAS* had been knocked out using CRISPR. In these wild-type RAS knockout lines, RMC-8839 alone completely inhibited pERK levels (Fig. 5B; Supplementary Fig S15). The loss of wild-type RAS isoforms did not affect growth in adherent cultures (Supplementary Fig. S16A), but consistent with the isogenic MIA PaCa2 models, we found that NCI-H1355 cells lacking wild-type RAS had a substantial lag in their ability to grow as spheroids (Supplementary Fig. S16B). Collectively, these data demonstrate that *KRAS*^G13C^ cell lines have a highly variable response to mutant-selective RAS(ON) inhibition, likely reflecting a variable dependence on wild-type RAS activation under both basal and inhibitor-treated conditions.

### Combination of RMC-8839 and RAS(ON) Multiselective Inhibitors Suppresses Tumor Growth

Although a RAS(ON) multiselective inhibitor can completely suppress MAPK signaling in cell lines with both wild-type and KRAS^G13C^ activation (Fig. 5A, NCI-H1355 and TOV-21G), the high concentrations used *in vitro* are not directly translatable to animal models (29). Furthermore, we have previously shown that in preclinical models, single-agent RAS(ON) multiselective inhibitors have less pronounced antitumor activity in tumors harboring codon 13 mutations compared with those with codon 12 mutations (28). We hypothesized that a combination of RMC-8839 with a RAS(ON) multiselective inhibitor may enhance antitumor activity in KRAS^G13C^ mutant tumors by maximally suppressing KRAS^G13C^ via covalent modification and inhibiting basal and adaptive wild-type RAS(ON) signaling. *KRAS*^G13C^ cell lines were treated with a matrix of concentrations of RMC-8839 and RMC-7977, and the pERK levels or viable cell numbers were measured after 4 hours or 5 days of treatment, respectively. Across the range of concentrations tested, the RAS(ON) doublet combination of RMC-8839 and RMC-7977 led to greater pathway suppression and inhibition of cell proliferation compared with either single agent (Fig. 6A and B). The benefit of adding RMC-7977 to RMC-8839 was most pronounced in the cell lines with submaximal pERK suppression by the mutant-selective RAS inhibitor (NCI-H1355, TOV-21G). A similar combination benefit was observed when the EGFR inhibitors gefitinib and erlotinib or the SHP2 inhibitor RMC-4550 were added in combination with RMC-8839, consistent with both wild-type and KRAS^G13C^ signaling contributing to cell growth (Supplementary Fig. S17).

**Figure 6.**
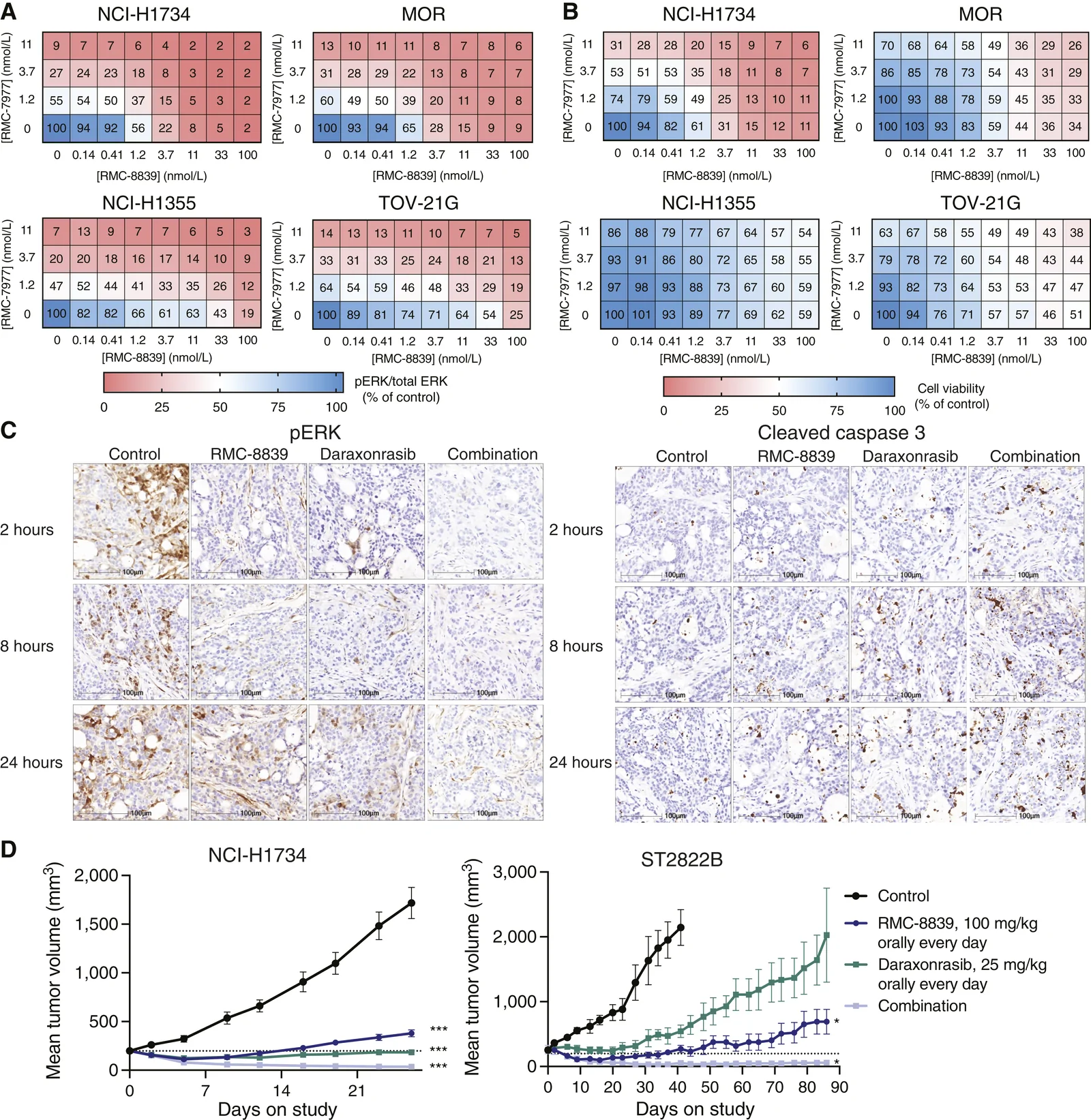
RMC-8839 and multiselective RAS(ON) inhibitor combinations increase the depth and durability of tumor regressions. *KRAS*^G13C^-mutant cancer cell lines were treated with a matrix of RMC-8839 and RMC-7977 combinations, and (**A**) pERK levels relative to total ERK levels were assessed after 4 hours of treatment, or (**B**) cell viability was assessed after 5 days of treatment. Mice bearing NCI-H1734 CDX tumors were treated with a single dose of either a single agent or the combination and (**C**) tumor levels of pERK or cleaved caspase 3 were assessed by IHC. **D,** Mice bearing NCI-H1734 CDX tumors were treated daily as indicated, and tumor volume was measured for 28 days (*n* = 9 per group, mean ± SEM), or ST2822B PDX tumors were treated daily with the indicated single-agent or combination treatment, and tumor volume was measured for 86 days (*n* = 3 per group, mean ± SEM). *, *P* < 0.05; ***, *P* < 0.001 two-way repeated measures ANOVA compared to the control on the last treatment day of the control group.

To explore this combination *in vivo*, we used RMC-8839 and the investigational RAS(ON) multiselective inhibitor daraxonrasib in the NCI-H1734 xenograft model. Although a single oral dose of daraxonrasib or RMC-8839 initially suppressed tumor *DUSP6* mRNA levels, reactivation of the pathway was observed by 24 hours (Supplementary Fig. S18A). Combination treatment with both agents resulted in sustained RAS pathway suppression for at least 24 hours. Consistent with the *DUSP6* expression, tumor IHC showed that the combination treatment could achieve greater suppression of pERK levels and increased caspase 3 cleavage over 24 hours compared with daraxonrasib or RMC-8839 alone (Fig. 6C; Supplementary Fig. S18B). In a 28-day tumor growth study in NCI-H1734 xenograft tumors, both RMC-8839 and daraxonrasib treatment suppressed tumor growth; however, deep and durable tumor regressions were observed only with the combination (Fig. 6D). A similar combination benefit was also seen in the *KRAS*^G13C^ NSCLC ST2822B PDX model, with the combination driving durable tumor regressions that persisted for the entire 86-day treatment. The combination of daraxonrasib and RMC-8839 was also well tolerated, with no body weight loss (Supplementary Fig. S18C). In summary, the RAS(ON) doublet combination of a RAS(ON) G13C-selective and RAS(ON) multiselective inhibitor resulted in strong pathway suppression and deep and durable tumor regressions in *KRAS*^G13C^ mutant xenograft models.

## Discussion

Adagrasib and sotorasib have demonstrated the clinical benefit of selectively targeting oncogenic KRAS^G12C^ proteins in NSCLC. KRAS^G12D^-selective inhibitors are now being tested in clinical trials (42), but mutant-selective inhibitors have not yet been developed for the many other KRAS variants found in human cancers. The underlying mechanism of oncogenic activation, elevated KRAS(ON) signaling, is conserved for all KRAS oncoproteins, yet the biophysics, biochemistry, genomics, and cell biology diverge significantly for many KRAS mutants, suggesting that mutant-specific treatment strategies may be necessary to achieve maximal RAS pathway suppression and optimal antitumor activity.

We profiled the biochemical properties of KRAS proteins with common mutations across the hotspot codons of 12, 13, and 61. KRAS^G13X^-mutant proteins have robust intrinsic and GAP-stimulated GTP hydrolysis compared with codon 12 and codon 61 mutants, accompanied by a slightly increased propensity for spontaneous nucleotide exchange that falls short of the codon 146 “hyperexchange” mutants. Consistent with the biochemical findings, isogenic cell lines demonstrated that KRAS^G13X^ has reduced oncopotency compared with equivalent KRAS^G12X^ mutants both *in vitro* and *in vivo*. We also found substantial differences between specific amino acid substitutions within the same codon; for example, KRAS^G12R^ is exceptionally disruptive to intrinsic and GAP-stimulated GTP hydrolysis, much more so than other G12X mutations. Further differences were also seen within the Q61X mutants, with most mutants (Q61K, Q61L, and Q61R) having slow intrinsic GTP hydrolysis (t_1/2_ > 24 hours at 37°C) and Q61H having markedly greater hydrolysis activity (t_1/2_ ∼ 7 hours at 37°C) that is more similar to G12V. The mutant-specific differences may be particularly relevant for pan-mutant KRAS inhibitors, which share a common binding site in the SWII pocket of GDP-bound KRAS(OFF). Although the pan-KRAS(OFF) inhibitors have similar biochemical affinity for all KRAS mutants, there is markedly greater cellular activity in wild-type, G12D, G12C, G12V, and Q61H KRAS compared with G12R, Q61K, Q61L, and Q61R KRAS (24, 43). The concordance between mutants with weak biochemical hydrolysis activity (G12R, Q61K, Q61L, and Q61R) and weak cellular potency is strongly suggestive that KRAS(OFF) inhibitor potency is limited by the accessibility of intracellular GDP-bound KRAS.

Probing further into biological differences between KRAS mutations, we established that codon 13 RAS mutations are significantly more likely to co-occur with *NF1* loss-of-function and *BRAF* class II/III gain-of-function mutations than the equivalent codon 12, and the introduction of these mutations enhances the oncopotency of *KRAS*^G13X^ in isogenic models. It is noteworthy that both *NF*1 loss-of-function and *BRAF* class II/III mutations are only mildly transforming (37, 44), in contrast to the much more strongly activating *BRAF* class I mutations that have been previously shown to demonstrate mutual exclusivity with *RAS* mutations, including *KRAS*^G13X^ mutations, in human tumors (39). The results, therefore, suggest a cooperative role in cellular transformation wherein both *KRAS*^G13X^ and *NF1* or *BRAF* class II/III are largely insufficient as single mutations to drive tumorigenesis, but the combination of the two alterations drives tumor growth. *NF1* and class III *BRAF* mutations are also expected to increase cellular dependence on wild-type RAS (37, 44), suggesting that codon 13 *KRAS*-mutant cancers rely on both mutant and wild-type RAS signaling.

RMC-8839 is the first reported drug-like covalent inhibitor of G13C-mutant RAS and was built using the CypA tricomplex platform that has previously yielded multiple RAS(ON) mutant- and multiselective inhibitors. RMC-8839 is a potent and selective inhibitor that covalently modifies the oncogenic G13C residue and inhibits mutant RAS(ON) while sparing wild-type signaling. However, potent and selective KRAS^G13C^ inhibition was not sufficient to extinguish RAS signaling in all *KRAS*^G13C^-mutant cell lines. Increased reliance on wild-type RAS is consistent with the comutation (Fig. 2D) and isogenic cell line (Fig. 2E) data demonstrating that *KRAS*^G13X^ mutations are not sufficiently transforming and may need concurrent mechanisms of pathway activation, as discussed above. Intriguingly, there was not a clear correlation between RMC-8839 sensitivity and co-occurring *NF1* or *BRAF* mutations, suggesting a role for alternative mutations or nongenomic mechanisms of activation of wild-type RAS in *KRAS*^G13C^-mutant cancer cells. Although it has been established that oncogenic RAS signaling desensitizes cells to signaling from RTKs (45), the reduced oncopotency of codon 13 RAS mutations may result in an increased dependence on RTK-driven wild-type RAS activation. There is precedent for *KRAS*^G13D^-mutant colorectal cancers having an increased dependence on RTK signaling. Clinical trials of cetuximab in *KRAS*-mutant colorectal cancer have found that although patients with codon 12 mutant tumors derive no benefit from EGFR inhibition, patients with KRAS^G13D^-mutant colorectal cancers may benefit from cetuximab treatment (46, 47).

The attenuated response to single-agent RMC-8839 we observed in some KRAS G13C preclinical models suggests that a combination therapeutic strategy may be required for maximal antitumor activity. Indeed, the RAS(ON) doublet combination of a RAS(ON) mutant-selective inhibitor, RMC-8839, with a RAS(ON) multiselective inhibitor, either RMC-7977 or daraxonrasib, enhanced RAS pathway suppression by cotargeting mutant and wild-type RAS(ON) proteins, resulting in complete RAS pathway suppression and maximal antitumor response. The accompanying work by McDaid and colleagues (48) suggests that in addition to increased reliance on wild-type RAS, the unique biology and co-occurring mutations observed in KRAS codon 13 mutant cancers may also render them sensitive to out-of-pathway and standard-of-care combinations with mutant-selective RAS inhibition. Additional work may therefore be needed to identify the appropriate biomarkers to direct patients to the most effective combination treatment regimens.

After decades without a targeted therapy for the treatment of RAS-mutant cancers, there is an ever-increasing number of agents being reported in the literature, tested in clinical trials, and approved by regulatory bodies. The emerging compounds, by virtue of their unique structures and binding sites, will have distinct profiles for ON versus OFF-state binding, mutant-selective versus concurrent mutant/wild-type targeting, and KRAS-selective versus RAS multiselective. The data presented here have several implications for the successful deployment of RAS inhibitors for the treatment of KRAS-mutant cancers. Specifically, the slow hydrolysis rate of G12R and Q61 mutants leads to highly GTP-bound KRAS and may require potent RAS inhibitors targeting the ON state. Additionally, *KRAS*^G13C^-mutant cancers may not all respond to single-agent mutant-selective RAS inhibition due to the increased frequency of in-pathway comutations, and *KRAS*^G13X^-mutant cancers, as a class, may require cotargeting of wild-type and mutant RAS to completely suppress KRAS signaling. This work highlights how a detailed understanding of RAS inhibitors, the underlying biochemical properties of specific RAS mutations, and an appreciation of the ways in which tumor biology differs across RAS-mutant genotypes can inform therapeutic strategies for clinical evaluation in patients with RAS-addicted cancers.

## Methods

### Protein Expression and Purification

His_6_-TEV-KRAS4B (residues 1–169), His_6_-TEV-CypA (full-length), His_6_-TEV-AviTag-CypA (full-length), and His_6_-TEV-RASA1 (residues 714–1047) were expressed from a pET28 vector in BL21 (DE3) *Escherichia coli*. His_6_-MBP-TEV-NF1 (residues 1198–1530 of NF1 isoform 2) was expressed from a pSYNO-1 vector in BL21 (DE3) *E. coli*. Cell pellets were lysed by sonication in lysis buffer [50 mmol/L Tris-HCl (pH 8.0), 500 mmol/L NaCl, 1 mmol/L phenylmethylsulfonylfluoride, 1 mmol/L Tris (2-carboxy-ethyl)-phosphin-HCl], and the recombinant proteins were isolated with Ni-NTA affinity chromatography. For KRAS, RASA1, and NF1, the His_6_ tag was cleaved by overnight dialysis [50 mmol/L Tris-HCl (pH 8.0), 500 mmol/L NaCl] at 4°C in the presence of His-tagged TEV protease (1:20 w/w). The cleaved tag and protease were removed by Ni-NTA chromatography, and the KRAS proteins were further purified by size exclusion chromatography on a Superdex 75 (26/600) column [20 mmol/L Tris-HCl (pH 8.0), 200 mmol/L NaCl]. For CypA, the His_6_ tag was cleaved by overnight dialysis [50 mmol/L Tris-HCl (pH 8.0), 300 mmol/L NaCl] at 4°C in the presence of His-tagged TEV protease (1:10 w/w). The cleaved tag and protease were removed by Ni-NTA chromatography. For avi-tagged CypA, the His_6_ tag was removed by overnight dialysis [50 mmol/L Tris-HCl (pH 8.0), 100 mmol/L NaCl, 7.5 mmol/L MgCl_2_] at 4°C in the presence of His-tagged TEV protease (1:10 w/w). The Avi tag was biotinylated by the addition of 1 mmol/L ATP, 0.5 mmol/L biotin, and 200 nmol/L BirA, followed by incubation at 18°C for 4 hours. The cleaved tag and protease were removed by Ni-NTA chromatography, and avi-CypA was further purified by Q anion exchange chromatography [20 mmol/L Tris-HCl (pH 8), 100–1,000 mmol/L NaCl] and size exclusion chromatography on a Superdex 75 (26/600) column [10 mmol/L HEPES-NaOH (pH 7.5), 150 mmol/L NaCl, 1 mmol/L MgCl_2_].

The purified KRAS proteins were loaded with GMPPNP by incubation in exchange buffer [40 mmol/L Tris-HCl (pH 7.5), 5 mmol/L dithiothreitol (DTT), 150 mmol/L (NH_4_)_2_SO_4_, 4 μmol/L ZnCl_2_] with 20 U of alkaline phosphatase per mg KRAS protein and a 5-fold molar excess of GMPPNP to KRAS proteins. After 4 hours of incubation at 30°C, MgCl_2_ was added to 5 mmol/L, and the excess nucleotide was removed using a HiTrap 5 mL desalting column pre-equilibrated in storage buffer [10 mmol/L HEPES-NaOH (pH 7.5), 150 mmol/L NaCl, 1 mmol/L MgCl_2_]. To load KRAS with either GTP or mantGDP, the KRAS proteins were incubated for 2 hours on ice in 12.5 mmol/L HEPES (pH 7.5), 75 mmol/L NaCl, 5 mmol/L EDTA, and a 10-fold molar excess of nucleotide to KRAS protein. The reaction was quenched by the addition of MgCl_2_ to 10 mmol/L and then buffer exchanged using Zeba Spin Desalting Columns (Pierce Biotechnologies). The GTP-loaded proteins were subsequently dialyzed in 12.5 mmol/L HEPES (pH 7.5) and 75 mmol/L NaCl overnight at 4°C.

### X-Ray Crystallography

Purified human CypA and KRAS^G13C^ bound to GMPPNP were combined in a 2:1 CypA/KRAS molar ratio in a buffer solution consisting of 12.5 mmol/L HEPES-NaOH (pH 7.3), 75 mmol/L NaCl, and 1 mmol/L MgCl_2_. Compounds were added from a 10 mmol/L DMSO stock to give a solution of 100 μmol/L KRAS^G13C^, 200 μmol/L CypA, and 300 μmol/L compound in a total volume of 1 mL. This mixture was incubated for 1 hour at 25°C, and the tricomplex was purified via gel filtration using a Superdex 75 10/300 GL column pre-equilibrated with a buffer consisting of 12.5 mmol/L HEPES-NaOH (pH 7.3), 75 mmol/L NaCl, and 1 mmol/L MgCl_2_. Fractions containing the tricomplex were pooled and concentrated to 15 mg/mL using an Amicon Ultra-4 30K centrifugal filter (MilliporeSigma). Eighty microliters of a screen composed of 0.1 mol/L 2-morpholin-4-ylethanesulfonic acid (MES), 18% to 29% PEG 3350 (increasing by 1% increments), and 0 to 0.14 mol/L NaCl (increasing by 0.02 mol/L increments) were dispensed into the wells of an MRC-2 crystallization plate. Well solution (0.3 μL) was mixed with 0.3 μL of the concentrated tricomplex in a sitting drop, and the plate was incubated at 18°C. Crystals grew overnight and were vitrified following cryoprotection via mother liquor supplemented with 25% glycerol.

X-ray diffraction datasets were collected at the Advanced Photon Source (APS-23-ID-B) at 100 K. Data were processed with XDS (49), and initial structures were determined via Phaser (50) using previously solved KRAS and CypA as molecular replacement search models. Ligand restraints were generated using AceDRG (51). The final structures were determined through iterative rounds of model building using Coot (52) and refinement using REFMAC5 from the CCP4 suite (53) and phenix.refine (54).

### Urea Denaturation and Pulse Proteolysis

KRAS proteins at 10 μmol/L in a buffer consisting of 12.5 mmol/L HEPES (pH 7.5), 75 mmol/L NaCl, 5 mmol/L MgCl_2_, and 30 μmol/L GMPPNP were incubated overnight at 22°C with concentrations of urea ranging from 0 to 7 mol/L. Thermolysin (Promega) was then added to 0.2 mg/mL and incubated for 1 minute at room temperature, then quenched by the addition of EDTA to 50 mmol/L and phosphoramidon to 20 μmol/L. The proteins were resolved by SDS-PAGE and stained with SimplyBlue Safe Stain (Invitrogen), followed by imaging and protein quantification by densitometry. The C_m_ and ΔG_unfolding_ were determined as described previously for HRAS (32).

### Nucleotide Exchange Kinetics

MantGDP-loaded KRAS proteins were diluted in 12.5 mmol/L HEPES (pH 7.5), 75 mmol/L NaCl, and 10 mmol/L MgCl_2_ and equilibrated to 37°C. Reactions were initiated by adding a 2,000-fold molar excess of GMPPNP relative to KRAS, and the mant fluorescence was monitored with excitation at 360 nm and emission at 450 nm. Fluorescence values were normalized and fit to a one-phase exponential decay to obtain a spontaneous nucleotide exchange rate constant.

### GTP Hydrolysis Measurements

Buffer consisting of 12.5 mmol/L HEPES (pH 7.5), 75 mmol/L NaCl, 1 mmol/L MgCl_2_, and 1 mmol/L DTT was pre-equilibrated to 37°C. Reactions were initiated by the addition of GTP-loaded KRAS proteins, and time points were quenched by heating to 80°C for 10 minutes. The quenched reactions were kept on ice or frozen at −20°C. The relative concentration of GTP at each time point was assessed by the GTPase-Glo assay (Promega) according to the manufacturer’s protocols. The GTP levels were normalized and fit to a one-phase exponential decay to obtain an intrinsic hydrolysis rate constant.

To assess GAP sensitivity, reactions were prepared as described above, and the indicated concentrations of the recombinant GAP domains of NF1 or RASA1 (p120) were added and incubated for 20 minutes. The fraction of GTP remaining was fit to a four-parameter sigmoidal concentration–response curve to obtain the effective concentration of GAP required to half-maximally stimulate hydrolysis (EC_50_).

### Cell Lines and Engineering

Cell lines obtained from ATCC were as follows: NCI-H1355 (H1355; ATCC, CRL-5865), NCI-H1734 (ATCC, CRL-5800), TOV-21G (ATCC, CRL-3577), MIA PaCa-2 (ATCC, CRL-1420), NCI-H1975 (ATCC, CRL-5908), U-2 OS (ATCC, HTB-96), and A-375 (A375; ATCC, CRL-1619). Cell lines from the European Collection of Animal Cell Cultures (ECACC) were as follows: MOR (84112312) and MOR/CPR (96042333). KKU-213 (CSC-C6856J) cells were obtained from Creative Bioarray. All cells were cultured in RPMI 1640 supplemented with 10% FBS and penicillin/streptomycin, except that RPMI 1640 was replaced with DMEM for MIA PaCa-2, KKU-213, and U-2 OS. Banked cell stocks were validated by short tandem repeat profiling and confirmed to be *Mycoplasma*-free by PCR (IDEXX BioAnalytics). Cells were kept for a maximum of 10 passages in culture before being discarded.

### Cellular RAS-RAF nanoBRET Assay

The RAF1 RBD (residues 51–149) was cloned into the pHTC vector (Promega) to incorporate a C-terminal HaloTag. Full-length wild-type or G13C KRAS4B was cloned into the pNLF1 vector (Promega) to incorporate an N-terminal nanoluciferase tag. U-2 OS cells were cotransfected with the nanoluciferase-tagged KRAS and HaloTag RAF1 RBD, and the nanoBRET signal was monitored as described previously (28). For expression of NF1, residues 1198 to 1551 of NF1 isoform 1 were cloned into p3xFLAG7.1 and transfected at the indicated plasmid ratios.

### Analysis of Comtations in KRAS-Mutant Tumors

Foundation Medicine Insights version MI20240507 (May 7, 2024, release) was queried for patients with colorectal cancer or NSCLC and the indicated *KRAS* mutations. *KRAS*^G12X^ encompassed the mutations G12D, G12C, G12V, G12R, G12A, G12S, G12F, G12L, G12I, and G12E, and *KRAS*^G13X^ encompassed G13D, G13C, G13V, G13R, G13E, and G13F on the threshold of greater than or equal to 10 patients in the database. To analyze comutations in each *KRAS*-mutant population, genes were filtered to remove anything with less than five counts in either group. Statistical significance for co-occurring mutations was determined by a *χ*^2^ test followed by the Benjamini–Hochberg procedure.

### Spheroid Growth Assays

MIA PaCa-2 cells were engineered via CRISPR knock-in to introduce homozygous *KRAS*^G13C^, *KRAS*^G13D^, or *KRAS*^G12D^. Cells were electroporated with CRISPR-Cas9 ribonucleoprotein containing multiple *NF1*-targeting sgRNAs to knock out *NF1*. To introduce *BRAF*^D594G^, cells were transduced with lentiviral particles containing an EF1A promoter-driven *BRAF* mutant. The MIA PaCa-2 or NCI-H1355 cell lines were seeded at 1,000 cells per well in an ultralow attachment round-bottom plate, centrifuged at 1,000 × *g* for 10 minutes to settle the cells, and incubated for 24 hours to allow for spheroid formation. The relative spheroid area was subsequently monitored using an Incucyte live-cell imager (Sartorius).

### Plate-Based Cellular pERK Assay

Cell lines were seeded at 30,000 cells per well in 96-well plates and allowed to adhere overnight. Compounds were added, and cells were incubated for 4 hours at 37°C, then lysed and assayed for total ERK1/2 and phospho-(Thr202/Tyr204; Thr185/Tyr187)-ERK 1/2 using Meso Scale Diagnostics according to the manufacturer’s protocols.

### Cellular Viability Assays

The NCI-H1975 and *KRAS*^G13C^-mutant cell lines were seeded at 2,500 or 5,000 cells per well in an ultralow attachment round-bottom plate, centrifuged at 1,000 × *g* for 10 minutes to settle the cells, and incubated for 24 hours to allow for spheroid formation. Compounds were added, and the cells were incubated for a further 5 days. Cell viability was assessed by CellTiter-Glo 3D reagent (Promega), plotted relative to DMSO controls, and then fit to a four-parameter sigmoidal concentration–response curve. The *BRAF*^V600E^ cell line A375 was seeded at 125 cells per well in 384-well tissue culture–treated plates, allowed to adhere overnight, and then treated with compound for 5 days. Cell viability was assessed by CellTiter-Glo 2.0 reagent, plotted relative to DMSO controls, and then fit to a four-parameter sigmoidal concentration–response curve.

### Western Blotting

Cell lines were seeded at 1 million cells per well in six-well plates and incubated overnight. Compounds were added and incubated for either 4 or 24 hours (as indicated), then washed with PBS and lysed in NP-40 lysis buffer [50 mmol/L Tris-HCl (pH 7.4), 150 mmol/L NaCl, 1% NP-40, and 5 mmol/L EDTA] containing 2× HALT protease/phosphatase inhibitors (Pierce Biotechnology). The lysates were clarified by centrifugation, and the protein content was quantified by bicinchoninic acid (BCA) assay (Pierce Biotechnology). Protein-normalized samples were prepared, separated by SDS-PAGE, then transferred to nitrocellulose membranes and blocked in Intercept TBS buffer (LICORbio). The appropriate primary antibodies were added and incubated overnight in Intercept tris-buffered saline with 0.1% Tween (TBST) buffer at 4°C, followed by washing with TBST. Secondary antibodies in Intercept TBST buffer were incubated for 1 hour at ambient temperature, then washed and imaged on a LICOR Odyssey.

The antibodies used for immunoblots were: KRAS (MilliporeSigma, WH0003845M1), pan-RAS (Abcam, 108602), pERK (T202/Y204; Cell Signaling Technology, 9101), ERK (Cell Signaling Technology, 4696), GAPDH (Cell Signaling Technology, 97166 or 5174), and vinculin (Cell Signaling Technology, 13901). Goat IR680- or IR800-conjugated secondary antibodies were obtained from LICOR.

### Phospho-RTK Arrays

Cells were grown in the presence of RMC-8839 or DMSO, then rinsed with PBS and solubilized at 5 million cells per mL of lysis buffer prepared with protease inhibitors. The protein content of lysates was quantified by BCA assay (Pierce), and 1.4 mg of total protein was prepared in 1.5 mL of lysis buffer. The levels of phosphorylated RTKs were assessed by the Proteome Profiler Human Phospho-Kinase Array Kit (R&D Systems).

### RAF-RBD Pulldown and Mass Spectrometry

Five million cells (MOR, NCI-H1355, or TOV-21G) were seeded into 15-cm dishes and treated with 100 nmol/L of RMC-8839 or DMSO in triplicate. After 48 hours, the cells were harvested, and the RAS-GTP levels were assessed by the Active RAS Pulldown and Detection Kit (Thermo Fisher Scientific). The manufacturer’s protocol was followed, except that bound RAS/RAF was eluted in 100 mmol/L glutathione in PBS.

The relative levels of RAS isoforms in the input (total RAS) and RAF-RBD eluate (active RAS) fractions were assessed by mass spectrometry. The proteins were reduced and alkylated with DTT and iodoacetamide, then precipitated with acetone. The precipitated protein was resuspended in digestion buffer and digested with trypsin/Lys-C (Promega) overnight at 37°C. The digestion was desalted with Sep-Pak C18 (Waters), and internal standard (IS) peptides with a C-terminal heavy lysine or arginine were spiked into each sample to a final concentration of 50 nmol/L. Samples were analyzed by LC/MS on an Orbitrap Lumos (Thermo Fisher Scientific) using a 35-minute linear gradient and targeted MS2 acquisition. The levels of all wild-type RAS (peptide: LVVVGAGGVGK), G13C-mutant KRAS (peptide: LVVVGAGGVGK), or total KRAS (peptide: SFEDIHHYR), NRAS (peptide: SFADINLYR), and HRAS (peptide: SYGIPYIETSAK) in each fraction were assessed as relative peak areas of the light (analyte) to heavy (IS) peptide peak area using 4 to 6 fragment ions per precursor.

### Animal Studies

All mouse studies and procedures related to animal handling, care, and treatment complied with all applicable regulations and guidelines of the Institutional Animal Care and Use Committee at each facility with their approvals.

### NCI-H1734 Xenograft Studies

Studies were conducted at Pharmaron Inc. The NCI-H1734 tumor cells were maintained *in vitro* as a monolayer culture in RPMI 1640 medium supplemented with 15% heat-inactivated FBS at 37°C in an atmosphere of 5% CO_2_ in air. The cells growing in an exponential growth phase were harvested and counted for tumor inoculation. Female NOD-SCID 6- to 8-week-old mice from Beijing AniKeeper Biotech Co., Ltd. were used for these studies. Each mouse was inoculated subcutaneously in the right flank with NCI-H1734 tumor cells (1 × 10^7^) in 100 μL RPMI 1640/Matrigel mixtures (1:1 ratio) without serum for tumor development.

For the efficacy and pharmacokinetic (PK)/PD studies testing the single agent RMC-8839, treatments started at the time of randomization when the mean tumor volume reached 196 mm^3^ for the efficacy study. RMC-8839 was administered daily at the indicated doses via oral gavage. For the single-dose PK/PD and TE study, mice were administered with the indicated treatment when the mean tumor volume reached 438 mm^3^. At the indicated time points after a single dose of RMC-8839 at the indicated dose levels, plasma and tumor samples were collected for PK, PD, and TE analysis, respectively.

For the efficacy and PD studies testing RMC-8839, daraxonrasib (RMC-6236), and the combination of RMC-8839 and daraxonrasib, treatments started at the time of randomization when the mean tumor volume reached 198 mm^3^. In the single-dose PD study, mice were administered the indicated treatments when the mean tumor volume was in the range of 901 to 987 mm^3^. At the indicated time points after a single dose of RMC-8839, daraxonrasib, and the combination of RMC-8839 plus daraxonrasib at the indicated dose levels, plasma and tumor samples were collected for PD analysis.

All compounds were formulated in 10/20/10/60 (%v/v/v/v; DMSO/PEG400/Solutol HS15/water). RMC-8839 and daraxonrasib were coformulated in 10/20/10/60 (%v/v/v/v; DMSO/PEG400/Solutol HS15/water).

Tumors were measured in two dimensions using a digital caliper by an unblinded employee who was not involved in the design or analysis of the study results. The tumor volume in mm^3^ was calculated using the following formula: Volume = [(width)^2^ × length]/2, where width and length are measured in mm. Mice in the studies were weighed, and tumor measurements were collected 2 times a week.

The statistical significance of any differences in tumor volumes between the vehicle control and the treatment group(s) was assessed by two-way repeated measures ANOVA in GraphPad Prism software. *P* values less than 0.05 were considered significant. *, *P* < 0.05; **, *P* < 0.01; ***, *P* < 0.001.

### MIA PaCa-2 and Engineered MIA PaCa-2 Xenograft Studies

Tumor growth study of parental MIA PaCa-2 and engineered MIA PaCa-2 with G13C mutation was conducted at WuXi AppTec (Suzhou) Co., Ltd. The MIA PaCa-2 tumor cells were maintained *in vitro* as a monolayer culture in DMEM and 10% heat-inactivated FBS supplemented with 1% penicillin/streptomycin at 37°C in an atmosphere of 5% CO_2_ in air. The cells growing in an exponential growth phase were harvested and counted for tumor inoculation. Female NOD-SCID 6- to 8-week-old mice from Beijing Vital River Laboratory Animal Co., Ltd. were used for this study. Each mouse was inoculated subcutaneously at the right flank with MIA PaCa-2 tumor cells (1 × 10^7^) in 0.2 mL of PBS supplemented with Matrigel (1:1) for tumor development. Tumor growth was monitored twice a week.

The tumor growth study of the engineered MIA PaCa-2 with G13C mutation, either without or with NF1 knockout, was conducted at Translational Drug Development, LLC. The MIA PaCa-2 tumor cells were maintained *in vitro* as a monolayer culture in DMEM and 10% heat-inactivated FBS supplemented with 1% penicillin/streptomycin at 37°C in an atmosphere of 5% CO_2_ in air. The cells growing in an exponential growth phase were harvested and counted for tumor inoculation. Female NOD-SCID 6- to 8-week-old mice from Charles River Laboratories were used for this study. Each mouse was inoculated subcutaneously at the lower right flank with MIA PaCa-2 tumor cells (1 × 10^7^) in 0.1 mL of media supplemented with Matrigel (1:1) for tumor development. Tumor volume was monitored twice a week.

Tumors were measured in two dimensions using a digital caliper by an unblinded employee who was not involved in the design or analysis of study results. The tumor volume in mm^3^ was calculated using the following formula: Volume = [(width)^2^ × length]/2, where width and length are measured in mm. Mice in the studies were weighed, and tumor measurements were collected 2 times a week.

The statistical significance of any differences in tumor volumes between parental MIA PaCa-2 and engineered MIA PaCa-2 with the G13C mutation was assessed by two-way repeated measures ANOVA in GraphPad Prism software. *P* values less than 0.05 were considered significant. ***, *P* < 0.001.

The statistical significance of any differences in tumor volumes between engineered MIA PaCa-2 with the G13C mutation and those with NF1 knockout was assessed by two-way repeated measures ANOVA in GraphPad Prism software. *P* values less than 0.05 were considered significant. *, *P* < 0.05; **, *P* < 0.01.

### ST2822B Xenograft Studies

Studies were conducted at XenoSTART. Tumor fragments from the ST2822B PDX model, representing KRAS^G13C^ mutated human lung cancer, were harvested from host animals and implanted into the right flanks of female athymic nude 6- to 12-week-old mice, outbred and homozygous [Crl:NU(NCr)-Foxn1nu], Stock #: 490 from Charles River Laboratories; treatment started when the average tumor volume reached 253 to 255 mm^3^ for the study testing the single-agent RMC-8839. Treatment started when the average tumor volume reached 252 to 261 mm^3^ for the study testing RMC-8839, daraxonrasib (RMC-6236), and RMC-8839 plus daraxonrasib. RMC-8839 and daraxonrasib were administered via oral gavage daily at 100 and 25 mg/kg, respectively, using the formulation made of 10/20/10/60 (%v/v/v/v; DMSO/PEG400/Solutol/water). For the cotreatment of RMC-8839 and RMC-6236, RMC-8839 and RMC-6236 were coformulated in 10/20/10/60 (%v/v/v/v; DMSO/PEG400/Solutol/water) and administered via oral gavage. Tumors were measured in two dimensions using a digital caliper by an unblinded employee who was not involved in the design or analysis of study results. The tumor volume in mm^3^ was calculated using the following formula: Volume = [(width)^2^ × length]/2, where width and length are measured in mm. Mice in the studies were weighed, and tumor measurements were collected 2 times a week.

The statistical significance of any differences in tumor volumes between vehicle control and the treatment groups was assessed by two-way repeated measures ANOVA in GraphPad Prism software. *P* values less than 0.05 were considered significant. *, *P* < 0.05; **, *P* < 0.01.

### Mouse Plasma Bioanalysis

The plasma concentrations of RMC-8839 were determined using liquid chromatography-tandem mass spectrometry (LC/MS-MS). Briefly, whole blood samples were collected in K_2_EDTA tubes, stored on wet ice, and processed to plasma within 30 minutes of sample collection. An aliquot of plasma (20 μL) was transferred to a tube and quenched with a 10× volume of acetonitrile containing a cocktail of ISs. After thorough mixing and centrifugation, the supernatant was directly analyzed on a SCIEX 5500 triple quadrupole mass spectrometer equipped with a Shimadzu LC system. A Raptor Biphenyl 2.7 μm, 2.1 × 50 mm column was used with gradient elution for compound separation. RMC-8839 and IS (dexamethasone) were detected by positive electrospray ionization using multiple reaction monitoring (RMC-8839: m/z 516.9/322.3, and dexamethasone: m/z 393.4/373.0). The lower limit of quantification was 1 ng/mL. Bioanalysis was conducted at Pharmaron.

### PK Analysis

Concentrations reported as below the quantification limit were treated as zero for PK analysis. PK parameters were calculated by noncompartmental analysis of the concentration–time profiles using Phoenix WinNonlin (version 8.3, Certara). Apparent terminal elimination half-life (t_1/2_) values were calculated as ln(2)/k, where k represents the terminal elimination rate constant. Area under the concentration–time curve (AUC) values were estimated using a linear trapezoidal method. AUC_last_ values were calculated from the dosing time to the last quantifiable concentration. Maximum concentration (C_max_) was recorded as observed.

### *In Vivo* PD Analysis by *DUSP6* qPCR

RNA was extracted from 20 to 30 mg of tumor tissue using the RNeasy Plus Mini Kit (QIAGEN, 74134) with a High Throughput Tissue grinder, following the manufacturer’s protocol from the RNeasy Plus Mini Kit. Reverse transcription was carried out using the High-Capacity cDNA Reverse Transcription Kit (ABI, 4368814) according to the manufacturer’s protocol. The cDNA product was used for qPCR analysis using TaqMan Gene Expression Master Mix (ABI, 4369016). TaqMan primer probes specific to human *DUSP6* and *18S* (used as an internal control gene) were used to detect the levels from each sample in duplicates using a 10 μL final reaction volume in a 384-well clear optical reaction plate. For qPCR, Ct values of *DUSP6* and *18S* were obtained for analysis. The *DUSP6* Ct value was normalized to *18S*, and then the mean relative mRNA expression levels of each group were normalized to the vehicle control group.

### Tumor TE

Tumor TE was measured by LC/MS at IQ Proteomics LLC. Snap-frozen tumor samples were homogenized in 100 mmol/L N-(2-hydroxyethyl)piperazine-N'-3-propanesulfonic acid (EPPS; pH 8.1) and 8 mol/L urea, then fully denatured via the addition of SDS [1% final (w/v)]. Heavy KRAS^G13C^ was spiked into each lysate as an internal control to account for sample recovery. Samples were reduced with DTT, and cysteines were alkylated with iodoacetamide. Following alkylation, protein was isolated via SP3 cleanup. Isolated protein was resuspended in 100 mmol/L EPPS (pH 8.1). Samples were digested with LysC (overnight, room temperature) and trypsin (6 hours, 37°C). Digested samples were analyzed via Orbitrap Lumos instrument (Thermo Fisher Scientific) using a parallel reaction monitoring (PRM) assay targeting KRAS peptides (including modified G13C peptides). Relative engagement of the G13C peptide was determined as a percentage of the signal observed for that peptide compared with that of each control peptide. For each sample, the relative abundance of each quantified peptide was expressed as a percentage of the signal observed for the SFEDIHHYR IS peptide in that sample. Signal was further normalized by applying a scalar to all samples on a peptide-by-peptide basis such that the median abundance of each peptide across the vehicle-treated initial time point (t = 3 hours) samples was set to 100%.

### IHC

All tissues were fixed for up to 24 hours using 10% neutral buffered formalin and then moved to 70% ethanol for long-term storage. Formalin-fixed, paraffin-embedded sections (4 μm) were stained on the BOND-III Automated IHC Stainer using the manufacturer’s recommended settings. Anti-EpCAM rabbit mAb (Cell Signaling Technology, cat. #14452, clone: D9S3P) was used at 1:400 with citrate-based pH 9.0 heat-induced epitope retrieval. Anti–phospho-p44/42 MAPK (Erk1/2; Thr202/Tyr204) rabbit mAb (Cell Signaling Technology, cat. #4370, clone: D13.14.4E) was used at 1:1,000 with citrate-based pH 9.0 heat-induced epitope retrieval. Anti–cleaved caspase 3 (Asp175) rabbit polyclonal antibody (Cell Signaling Technology, cat. #9661) was used at 1:500 with citrate-based pH 9.0 heat-induced epitope retrieval. The BOND IHC Polymer Detection Kit (Leica, DS9800) was used to detect rabbit primary antibodies. 3,3′-Diaminobenzidine (DAB)-stained slides were scanned and digitized with a 3DHISTECH Pannoramic MIDI whole-slide scanner at 200× magnification.

## Supplementary Material

Table S1 Protein crystallography refinement parameters

Table S2 BRAF mutation classification and prevalence in KRAS G13X NSCLC

Table S3 BRAF mutation classification and prevalence in KRAS G13D CRC

Table S4 RTK Mutations in KRAS G12X and KRAS G13X Tumors

Table S5 Co-mutations in KRAS G13C-Mutant Cancer Cell Lines

Figure S1 Half-Lives of KRAS G13C and KRAS G12C in cancer cell lines by SILAC. (A) NCI-H1355 or (B) NCI-H358 cells were cultured in Heavy RPMI 1640 for SILAC for more than 5 doublings to ensure complete heavy isotope incorporation. Cells were then re-plated in light RPMI and cells were harvested at after various time points. The relative isotope abundance of the indicated mutant-specific or total KRAS peptide [(Total Area of Heavy Peptide)/(Total Area of Heavy Peptide + Total Area of Light Peptide)] was plotted for each sample.

Figure S2 Reaction progress curves for the measurement of spontaneous nucleotide exchange and intrinsic nucleotide hydrolysis. (A) The fluorescence of mantGDP-bound KRAS protein was normalized to initial and final values, then fit to a single phase exponential to obtain the spontaneous GDP exchange rate constant shown in Fig. 1F.(B) The log of the GDP exchange rate constants (Fig. 1F) and ΔG of unfolding relative to wild-type (Fig. 1E) for the destabilized RAS mutants were plotted to assess the correlation (r2 = 0.80). (C) GTP-loaded KRAS proteins were incubated, time points were quenched and the percent GTP remaining was plotted then fit to a single phase exponential to obtain the intrinsic GTP hydrolysis rate constant.

Figure S3 Arginine finger mutants of the NF1 catalytic domain abrogates the effect on KRAS:RAF RBD complexes in cells. Cells were co-transfected with KRAS and NF1 plasmid as described in Figure 2C, except that the NF1 plasmid contained the R1276Q mutation previously demonstrated to disrupt catalytic GAP activity.

Figure S4 Crystal structural characterization of KRAS G13C-CypA tri-complex induced by RMC-8839. KRAS G13C (green), RMC-8839 (blue), and CypA (wheat) tri-complex is shown. Interactions between CypA and RAS switch I (left) and RAS switch II (right) are indicated with dashed lines. The bottom panel shows the unbiased omit map (blue mesh) at 2.5 σ with electron density indicating a covalent bond between RMC-8839 and mutant residue G13C.

Figure S5 RMC-8839 is selective for KRAS G13C over other RAS-mutant NSCLC Cancer Cell Lines. NSCLC cancer cell lines were seeded in 3D spheroid format and treated with RMC-8839 for 5 days. The pEC50 for inhibiting cell viability is plotted for the cell lines containing each KRAS mutation and the mean selectivity for that mutant vs. G13C is indicated above each bar. The cell lines tested were G13C [NCI-H1734, NCI-H1355, MOR], G12C [NCI-H358, LU65, CALU1], G12D [T3M10, A427, SKLU1], G12V [NCI-H441, SW900, H2445], G12A/S [A549, NCI-H2009], G13D [NCI-H1944], wild-type [NCI-H2228, PC9]

Figure S6 RMC-8839 is selective for KRAS G13C over RAS-independent cells. NCI-H1734 (KRAS G13C) or RAS-independent A375 (BRAF V600E) cell lines were treated with RMC-8839 for 5 days and the cell viability was assessed. The potency of RMC-8839 is >1000x greater in NCI-H1734 than the RAS-independent A375 cell line.

Figure S7 RMC-8839 was well-tolerated at daily doses up to 100 mg/kg. Mice bearing NCI-H1734 tumors were treated once daily with the indicated oral dose and the mouse body weights were monitored for 28 days.

Figure S8 Suppression of MAPK Pathway Signaling at End of 28-day Study. (A) Mice bearing NCI-H1734 CDX tumors were treated once daily with the indicated oral doses of RMC-8839 for 28 days. Tumors were collected 8 hours after the final dose and pERK was assessed by IHC.

Figure S9 RMC-8839 effects on cellular signaling in RAS wild-type cell line. The wild-type RAS cell line NCI-H1975 was treated with the indicated concentration of RMC-8839 or RMC-7977 and lysed after 4 hours (left) or 24 hours (right) of treatment.

Figure S10 Complete covalent modification of KRAS G13C by RMC-8839 in the partially responsive cell lines H1355 and TOV-21G. The covalent modification of KRAS G13C was monitored by disappearance of the unmodified KRAS G13C peptide as assessed by protein digestion and mass spectrometry ([RMC-8839] = 100 nM, 4 hrs)

Figure S11 RMC-8839 effects on cellular signaling in KRAS G13C cell lines at later time points. The indicated cell lines were treated with RMC-8839 for 24 hours prior to lysis and evaluation of protein levels by immuno-blotting.

Figure S12 Basal Phospho-RTK Profiling of KRAS G13C-Mutant Cell Lines. KRAS G13C-mutant cell lines (NCI-H1355, KKU-213, TOV-21G, NCI-H1734, MOR, and MOR/CPR) or an EGFR-mutant control cell line (NCI-H1975) were lysed and subjected to membrane array (Proteome Profiler Human Phospho-RTK Array). Duplicate spots with detectable signals are indicated with the boxes.

Figure S13 Phospho-RTK Profiling of KRAS G13C-Mutant Cell Lines Treated with RMC-8839. KRAS G13C-mutant cell lines (NCI-H1355, KKU-213, TOV-21G, NCI-H1734, MOR, and MOR/CPR) or an EGFR-mutant control cell line (NCI-H1975) were treated with DMSO or RMC-8839 (100 nM) for 4, 24, or 48 hours then lysed and subjected to membrane array (Proteome Profiler Human Phospho-RTK Array). The relative levels of each phospho-RTK were assessed be densitometry normalized to the reference spots on each array. Only RTKs with detectable signal in at least one time point are plotted. ND = not detected in that sample.

Figure S14 Measurement of Total and Active (RAF-RBD Pulldown) RAS Proteins. NCI-H1355, TOV-21G, or NCI-H1734 cells were seeded and treated with RMC-8839 (100 nM) for 48 hours prior to lysis. The levels of total (RAF-RBD pulldown input) and active (RAF-RBD pulldown elution) RAS proteins were assessed by mass spectrometry relative to a heavy peptide standard.

Figure S15 Suppression of cellular pERK levels by RMC-8839 in cell lines with knockout of wild-type RAS. NCI-H1355 and TOV-21G cell lines, either parental or with knockout of wild-type NRAS, HRAS, and KRAS, were treated with RMC-8839 for 4 hours and pERK levels were assessed.

Figure S16 Loss of Wild-Type RAS Affects Spheroid but not Adherent Cell Growth in KRAS G13C-Mutant Cell Lines. (A) NCI-H1355 or TOV-21G parental or wild-type HRAS, NRAS, and KRAS knockout cells were seeded in adherent culture and the growth kinetics were assessed by imaging. (B) The NCI-H1355 parental or wild-type RAS knockout cells were seeded in the wells of an ultra-low attachment plate and the spheroid area was assessed by imaging. Neither the parental or wild-type RAS knockout TOV-21G cells grew as spheroids.

Figure S17 RTK and SHP2 Inhibitors Combine With RMC-8839 to Enhance Suppression of Cellular Proliferation. KRAS G13C-mutant cancer cell lines were treated with a matrix of RMC-8839 and RTK or SHP2 inhibitors and cell viability was assessed after 5 days of treatment.

Figure S18 Combination of RMC-8839 and daraxonrasib. (A) NCI-H1734 tumors excised at various times after a single dose of RMC-8839, daraxonrasib, or the combination were assessed for expression of DUSP6 mRNA (mean ± SEM, n=3). (B) Quantification of treated NCI-H1734 CDX tumors for phospho-ERK or cleaved caspase 3 (CC3) as assessed by IHC (see Fig. 6C) (C) The body weights of mice treated with vehicle control, RMC-8839 single agent (100 mg/kg po qd), RMC-6236 (25 mg/kg po qd), or the combination therapy were monitored for the 86-day treatment period (n=3, mean ± SEM).

## Data Availability

Data used to generate analyses and visualizations in this publication are available within the article and its Supplementary data files.
