# Supplementary material for: Selective Inhibition of KRASG13C Reveals an Increased Dependence on Wild-type RAS Isoforms in Codon 13 RAS-Mutant Cancers
Source: Cancer Discov. 2026 Jun 22;16(11):2256–73. doi: 10.1158/2159-8290.CD-25-0886 (PMC13725342; doi:10.1158/2159-8290.CD-25-0886)
Supplement: Supplementary file 1 [file cd-25-0886_figure_s10_suppsf10.pdf]

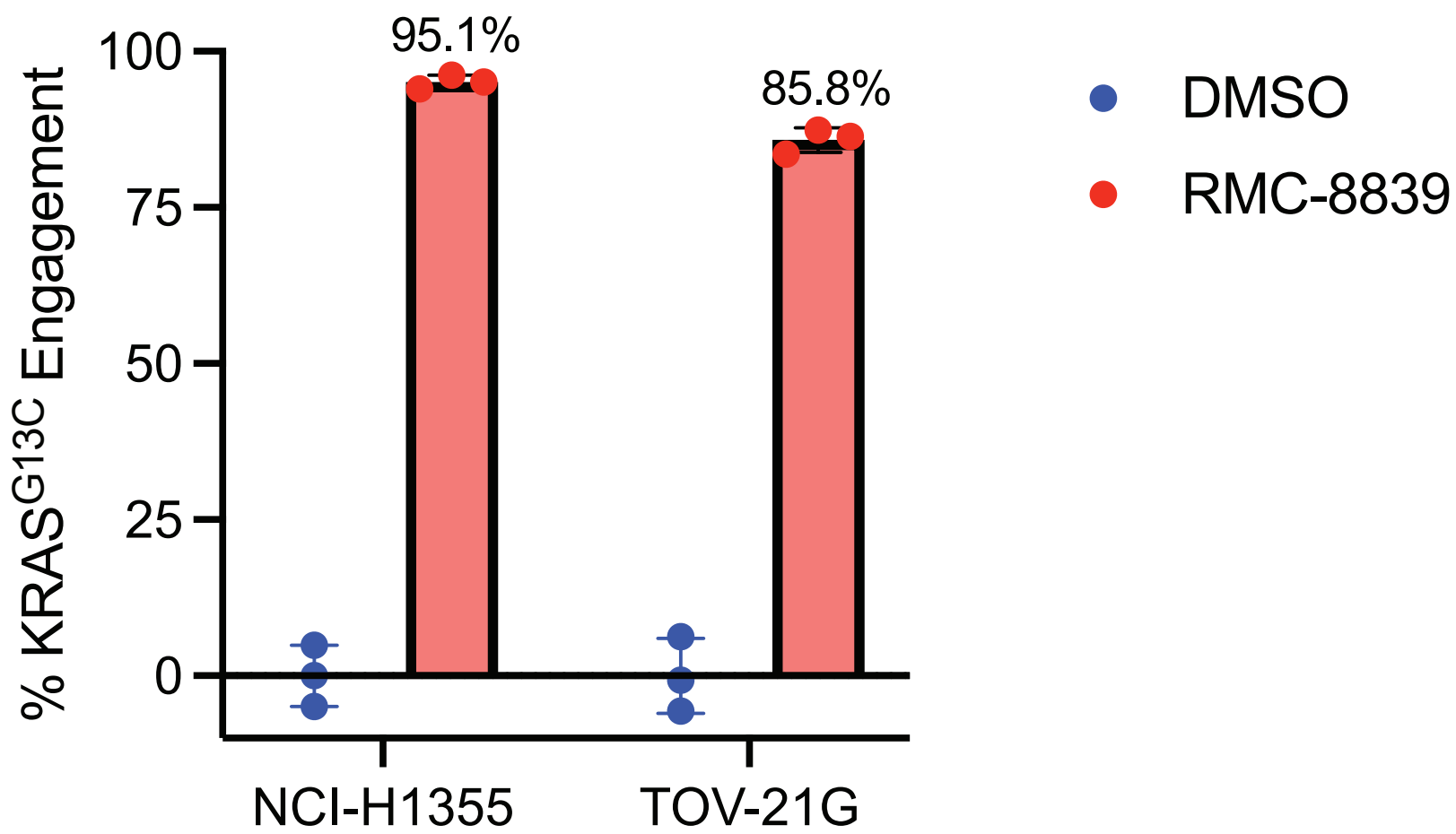

Supplementary Figure S10: Complete covalent modification of KRAS G13C by RMC-8839 in the partially-responsive cell lines H1355 and TOV-21G. The covalent modification of KRAS G13C was monitored by disappearance of the unmodified KRAS G13C peptide as assessed by protein digestion and mass spectrometry ([RMC-8839] = 100 nM, 4 hrs)
