# Supplementary material for: Selective Inhibition of KRASG13C Reveals an Increased Dependence on Wild-type RAS Isoforms in Codon 13 RAS-Mutant Cancers
Source: Cancer Discov. 2026 Jun 22;16(11):2256–73. doi: 10.1158/2159-8290.CD-25-0886 (PMC13725342; doi:10.1158/2159-8290.CD-25-0886)
Supplement: Supplementary file 2 [file cd-25-0886_figure_s11_suppsf11.pdf]

Figure S11

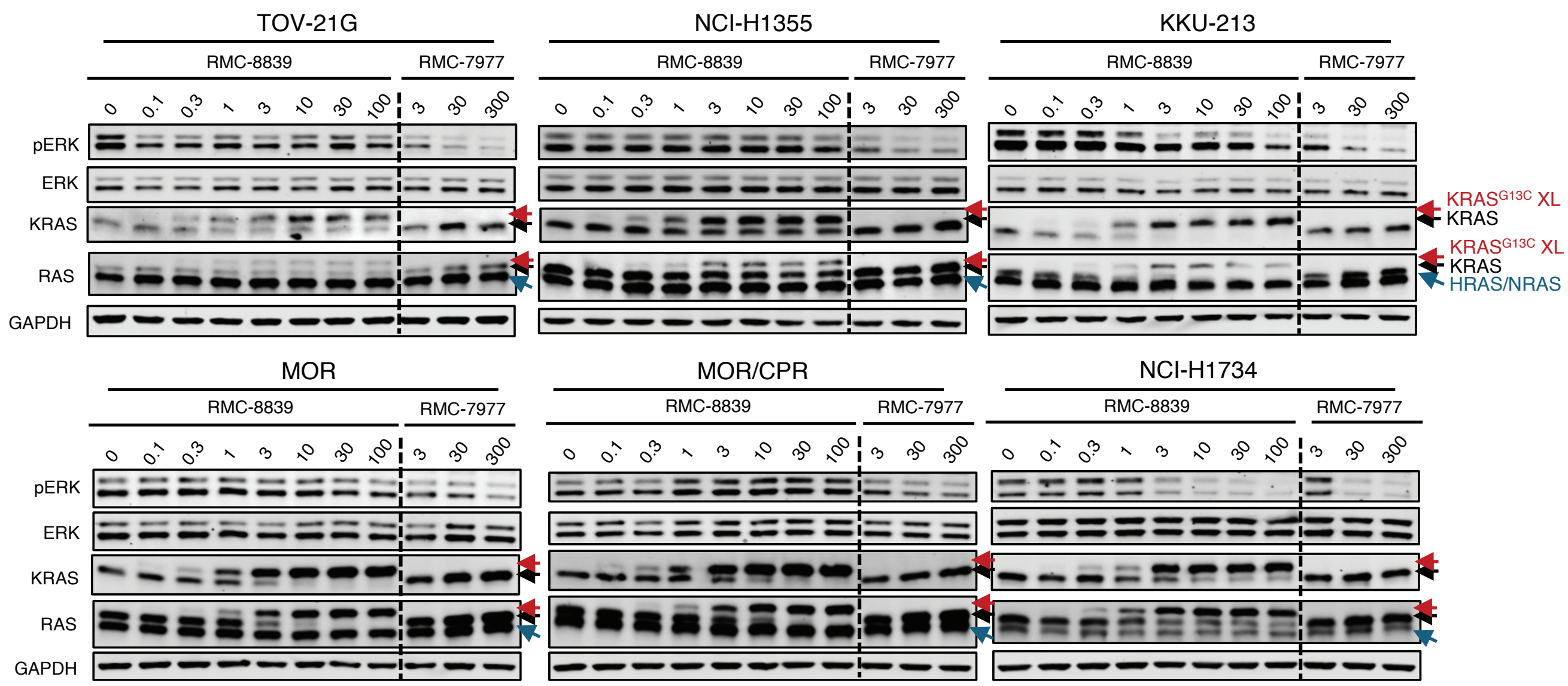

Supplementary Figure S11: RMC-8839 effects on cellular signaling in KRAS G13C cell lines at later time points. The indicated cell lines were treated with RMC-8839 for 24 hours prior to lysis and evaluation of protein levels by immuno-blotting.
