# Supplementary material for: Selective Inhibition of KRASG13C Reveals an Increased Dependence on Wild-type RAS Isoforms in Codon 13 RAS-Mutant Cancers
Source: Cancer Discov. 2026 Jun 22;16(11):2256–73. doi: 10.1158/2159-8290.CD-25-0886 (PMC13725342; doi:10.1158/2159-8290.CD-25-0886)
Supplement: Supplementary file 3 [file cd-25-0886_figure_s12_suppsf12.pdf]

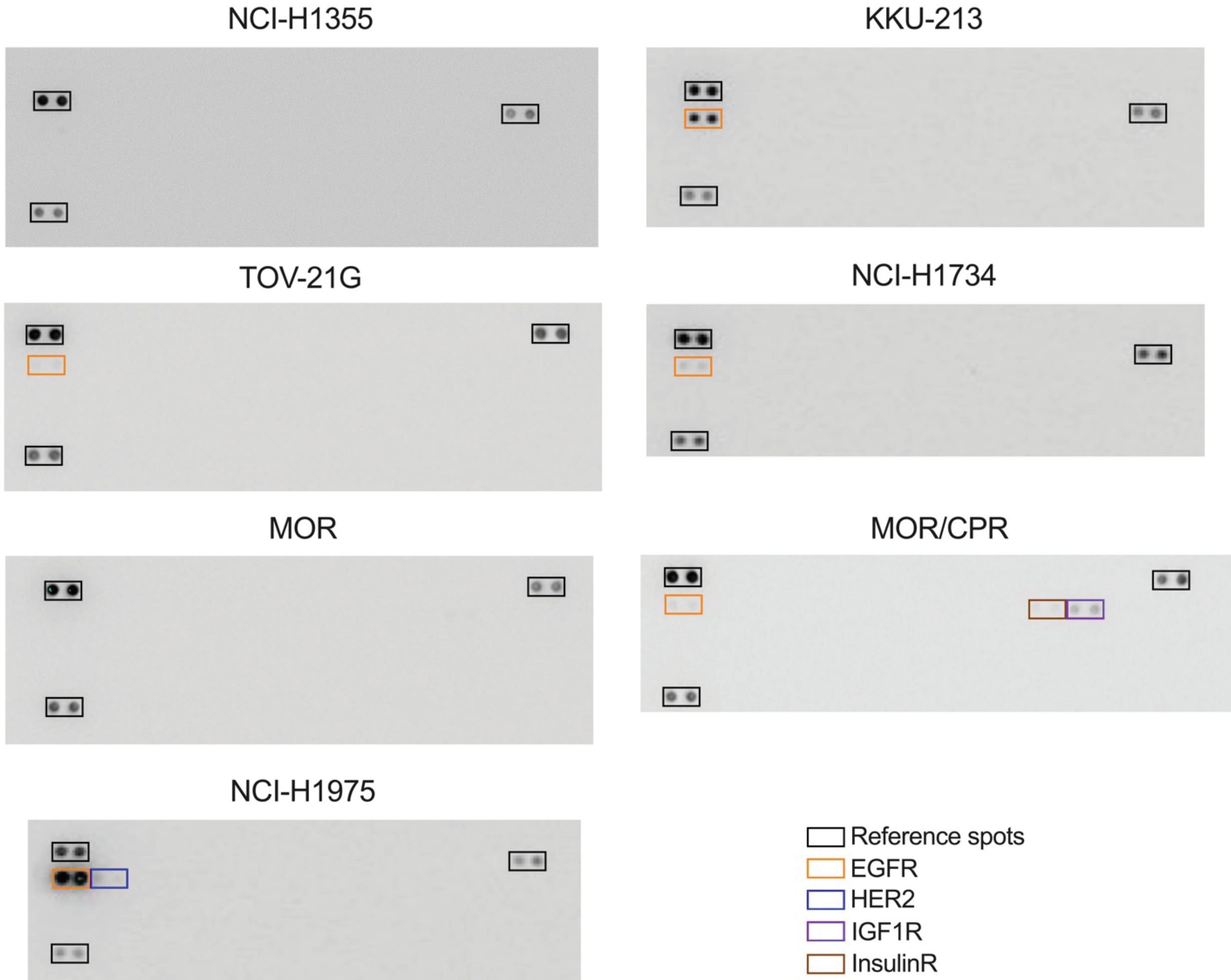

Supplementary Figure S12: Basal Phospho-RTK Profiling of KRAS G13C-Mutant Cell Lines. KRAS G13C-mutant cell lines (NCI-H1355, KKU-213, TOV-21G, NCI-H1734, MOR, and MOR/CPR) or an EGFR-mutant control cell line (NCI-H1975) were lysed and subjected to membrane array (Proteome Profiler Human Phospho-RTK Array). Duplicate spots with detectable signals are indicated with the boxes.
