# Supplementary material for: Selective Inhibition of KRASG13C Reveals an Increased Dependence on Wild-type RAS Isoforms in Codon 13 RAS-Mutant Cancers
Source: Cancer Discov. 2026 Jun 22;16(11):2256–73. doi: 10.1158/2159-8290.CD-25-0886 (PMC13725342; doi:10.1158/2159-8290.CD-25-0886)
Supplement: Supplementary file 4 [file cd-25-0886_figure_s13_suppsf13.pdf]

Figure S13

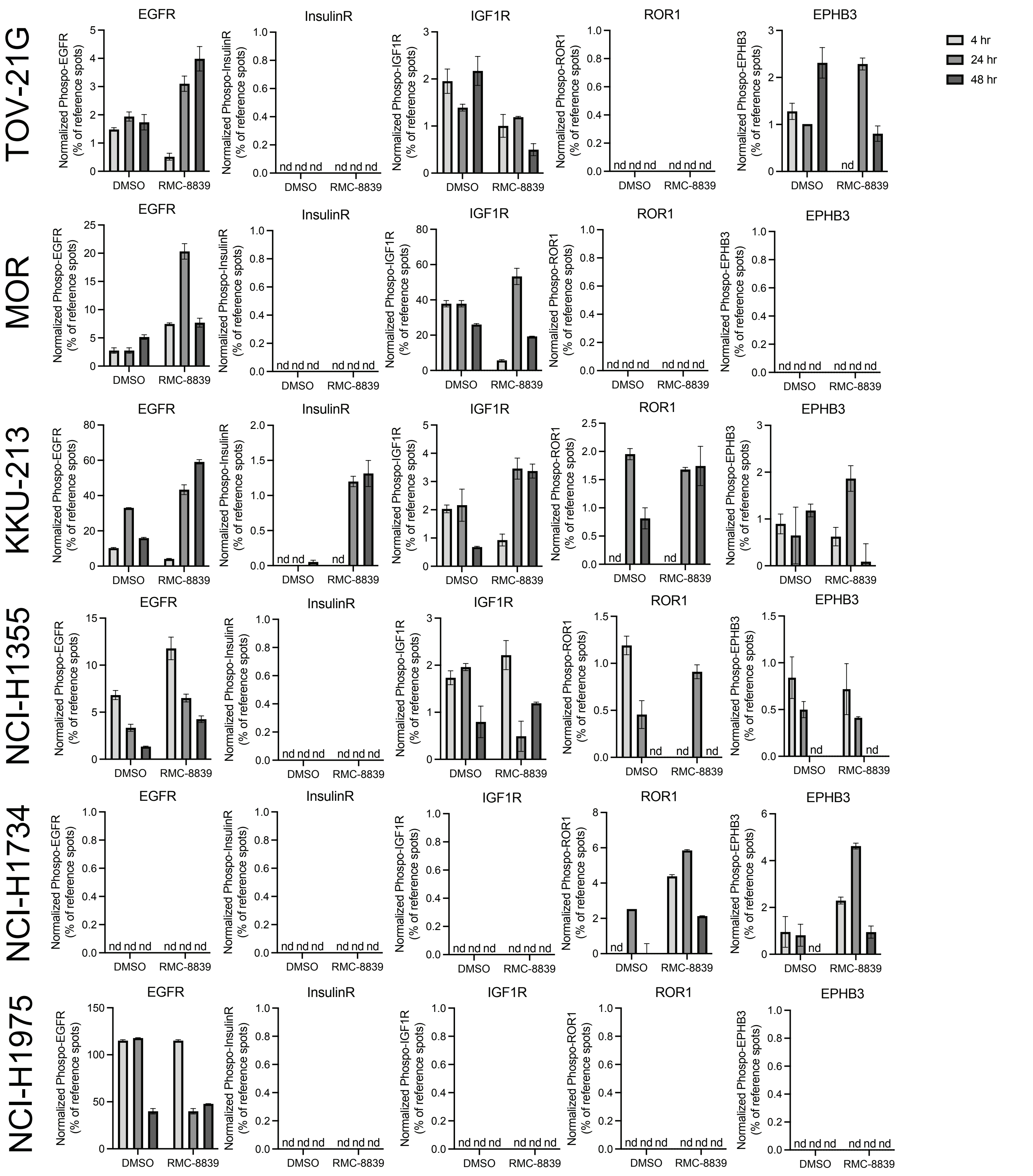

Supplementary Figure S13: Phospho-RTK Profiling of KRASG13C-Mutant Cell Lines Treated with RMC-8839. KRAS G13C-mutant cell lines (NCI-H1355, KKU-213, TOV-21G, NCI-H1734, MOR, and MOR/CPR) or an EGFR-mutant control cell line (NCI-H1975) were treated with DMSO or RMC-8839 (100 nM) for 4, 24, or 48 hours then lysed and subjected to membrane array (Proteome Profiler Human Phospho-RTK Array). The relative levels of each phospho-RTK were assessed be densitometry normalized to the reference spots on each array. Only RTKs with detectable signal in at least one time point are plotted. ND = not detected in that sample.
