# Supplementary material for: Selective Inhibition of KRASG13C Reveals an Increased Dependence on Wild-type RAS Isoforms in Codon 13 RAS-Mutant Cancers
Source: Cancer Discov. 2026 Jun 22;16(11):2256–73. doi: 10.1158/2159-8290.CD-25-0886 (PMC13725342; doi:10.1158/2159-8290.CD-25-0886)
Supplement: Supplementary file 5 [file cd-25-0886_figure_s14_suppsf14.pdf]

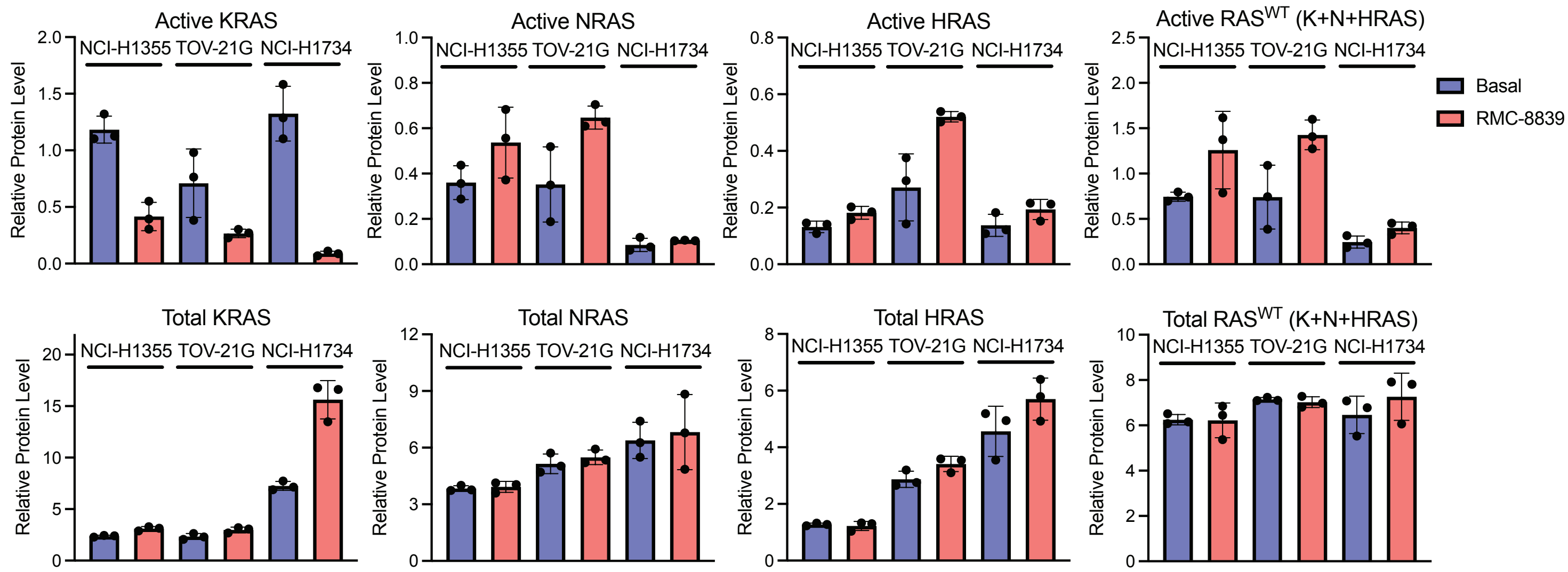

Supplementary Figure S14: Measurement of Total and Active (RAF-RBD Pulldown) RAS Proteins. NCI-H1355, TOV-21G, or NCI-H1734 cells were seeded and treated with RMC-8839 (100 nM) for 48 hours prior to lysis. The levels of total (RAF-RBD pulldown input) and active (RAF-RBD pulldown elution) RAS proteins were assessed by mass spectrometry relative to a heavy peptide standard.
