# Supplementary material for: Selective Inhibition of KRASG13C Reveals an Increased Dependence on Wild-type RAS Isoforms in Codon 13 RAS-Mutant Cancers
Source: Cancer Discov. 2026 Jun 22;16(11):2256–73. doi: 10.1158/2159-8290.CD-25-0886 (PMC13725342; doi:10.1158/2159-8290.CD-25-0886)
Supplement: Supplementary file 6 [file cd-25-0886_figure_s15_suppsf15.pdf]

# Figure S15

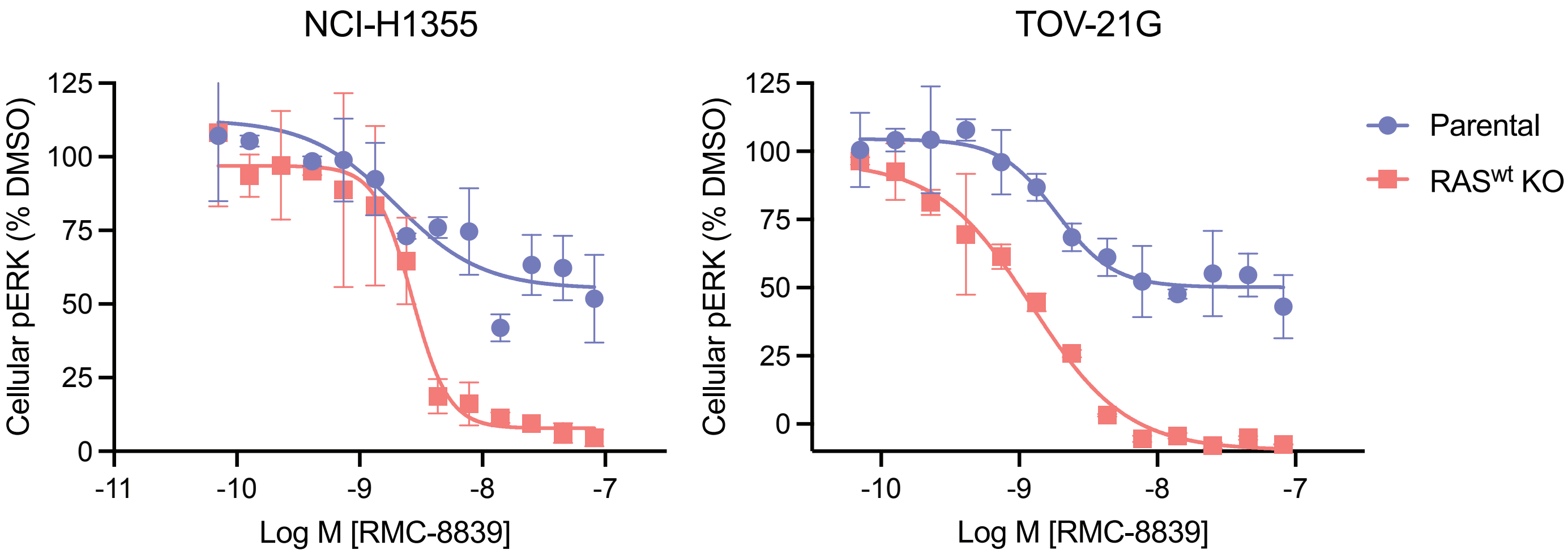


Supplementary Figure S15: Suppression of cellular pERK levels by RMC-8839 in cell lines with knockout of wild-type RAS. NCI-H1355 and TOV-21G cell lines, either parental or with knockout of wild-type NRAS, HRAS, and KRAS, were treated with RMC-8839 for 4 hours and pERK levels were assessed.
