# Supplementary material for: Selective Inhibition of KRASG13C Reveals an Increased Dependence on Wild-type RAS Isoforms in Codon 13 RAS-Mutant Cancers
Source: Cancer Discov. 2026 Jun 22;16(11):2256–73. doi: 10.1158/2159-8290.CD-25-0886 (PMC13725342; doi:10.1158/2159-8290.CD-25-0886)
Supplement: Supplementary file 7 [file cd-25-0886_figure_s16_suppsf16.pdf]

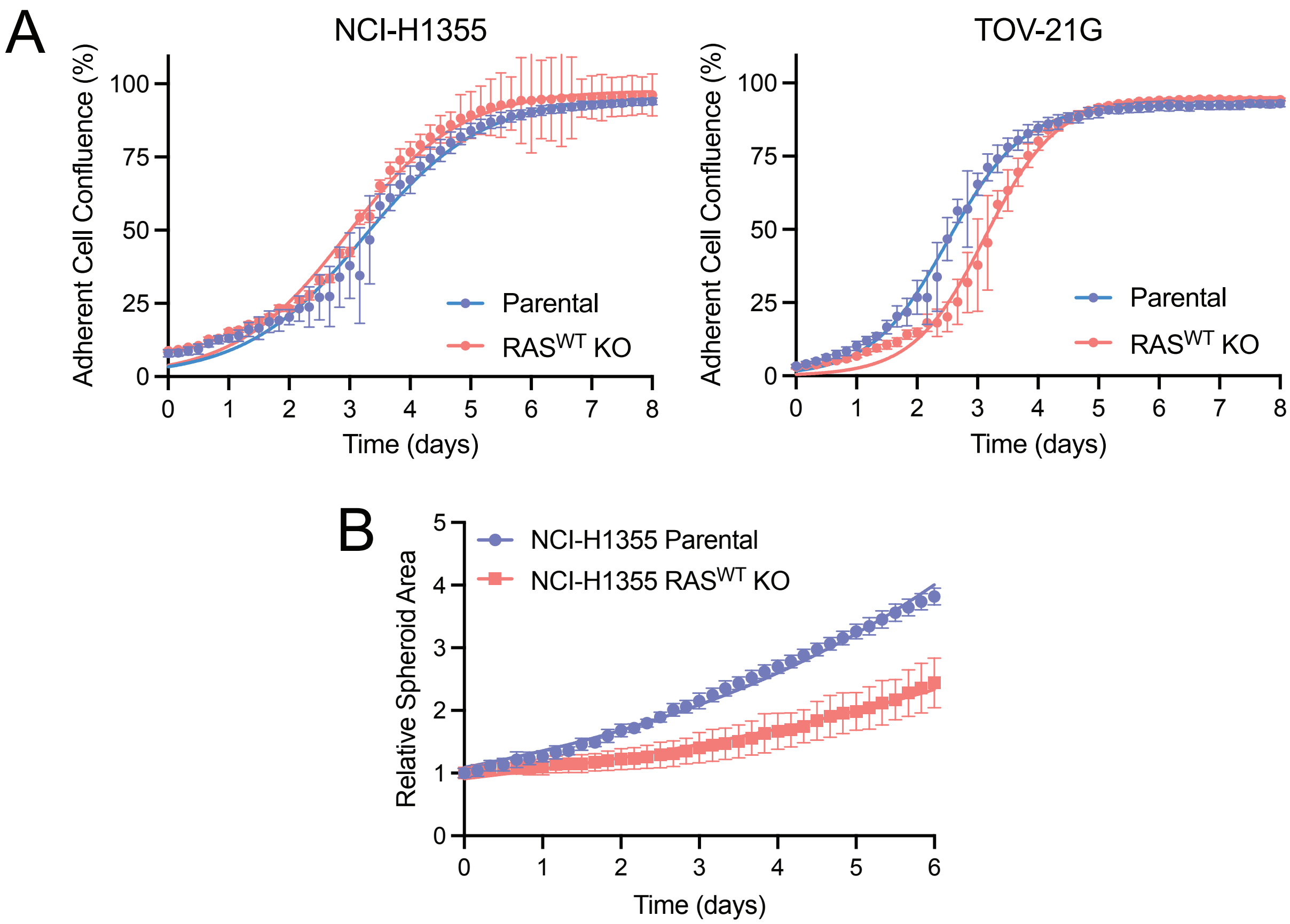

Supplementary Figure S16: Loss of Wild-Type RAS Affects Spheroid but not Adherent Cell Growth in KRAS G13C-Mutant Cell Lines. (A) NCI-H1355 or TOV-21G parental or wild-type HRAS, NRAS, and KRAS knockout cells were seeded in adherent culture and the growth kinetics were assessed by imaging. (B) The NCI-H1355 parental or wild-type RAS knockout cells were seeded in the wells of an ultra-low attachment plate and the spheroid area was assessed by imaging. Neither the parental or wild-type RAS knockout TOV-21G cells grew as spheroids.
