# Supplementary material for: Selective Inhibition of KRASG13C Reveals an Increased Dependence on Wild-type RAS Isoforms in Codon 13 RAS-Mutant Cancers
Source: Cancer Discov. 2026 Jun 22;16(11):2256–73. doi: 10.1158/2159-8290.CD-25-0886 (PMC13725342; doi:10.1158/2159-8290.CD-25-0886)
Supplement: Supplementary file 8 [file cd-25-0886_figure_s17_suppsf17.pdf]

Figure S17

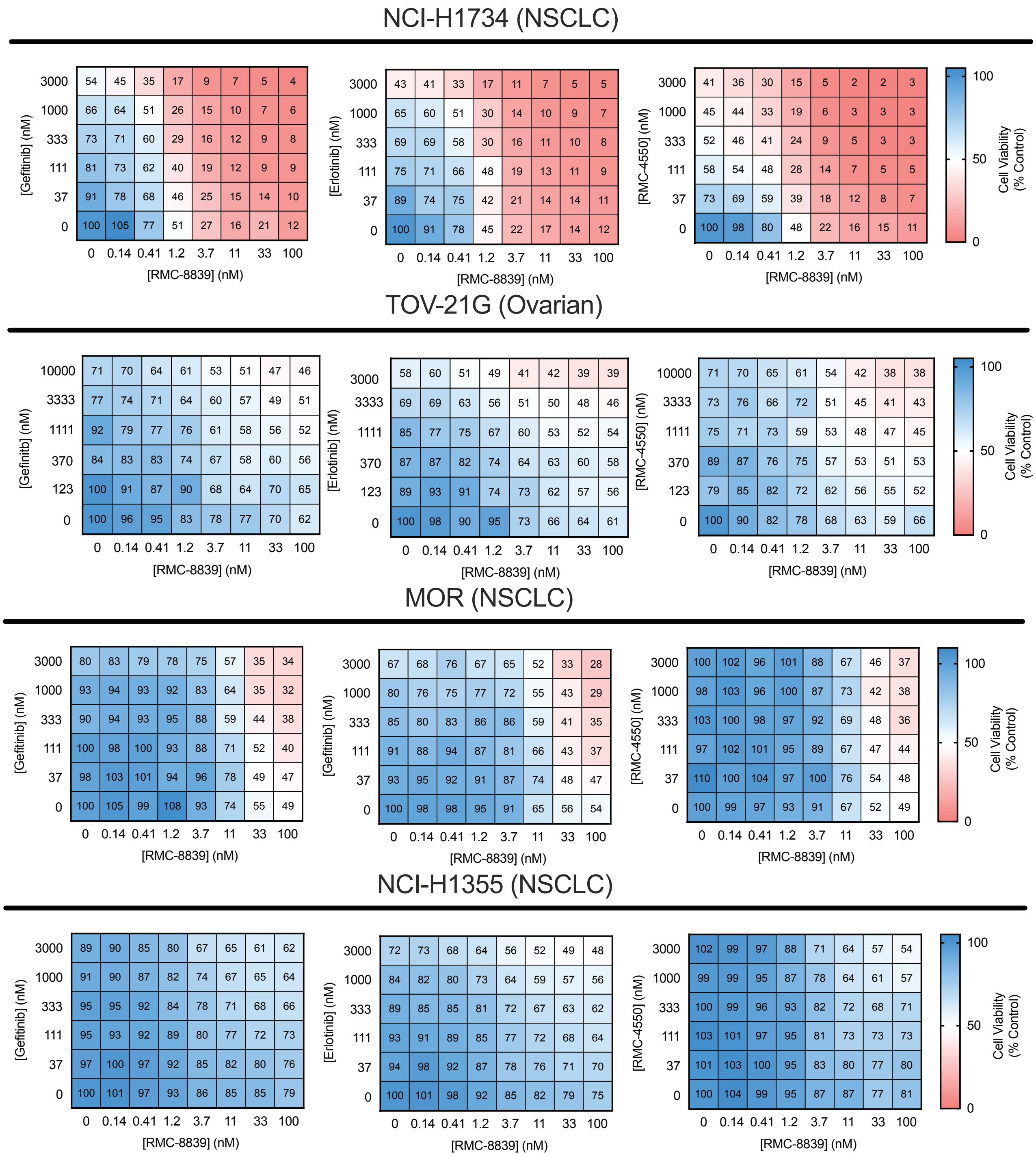

Supplementary Figure S17: RTK and SHP2 Inhibitors Combine With RMC-8839 to Enhance Suppression of Cellular Proliferation. KRAS G13C-mutant cancer cell lines were treated with a matrix of RMC-8839 and RTK or SHP2 inhibitors and cell viability was assessed after 5 days of treatment.
