# Supplementary material for: Selective Inhibition of KRASG13C Reveals an Increased Dependence on Wild-type RAS Isoforms in Codon 13 RAS-Mutant Cancers
Source: Cancer Discov. 2026 Jun 22;16(11):2256–73. doi: 10.1158/2159-8290.CD-25-0886 (PMC13725342; doi:10.1158/2159-8290.CD-25-0886)
Supplement: Supplementary file 9 [file cd-25-0886_figure_s18_suppsf18.pdf]

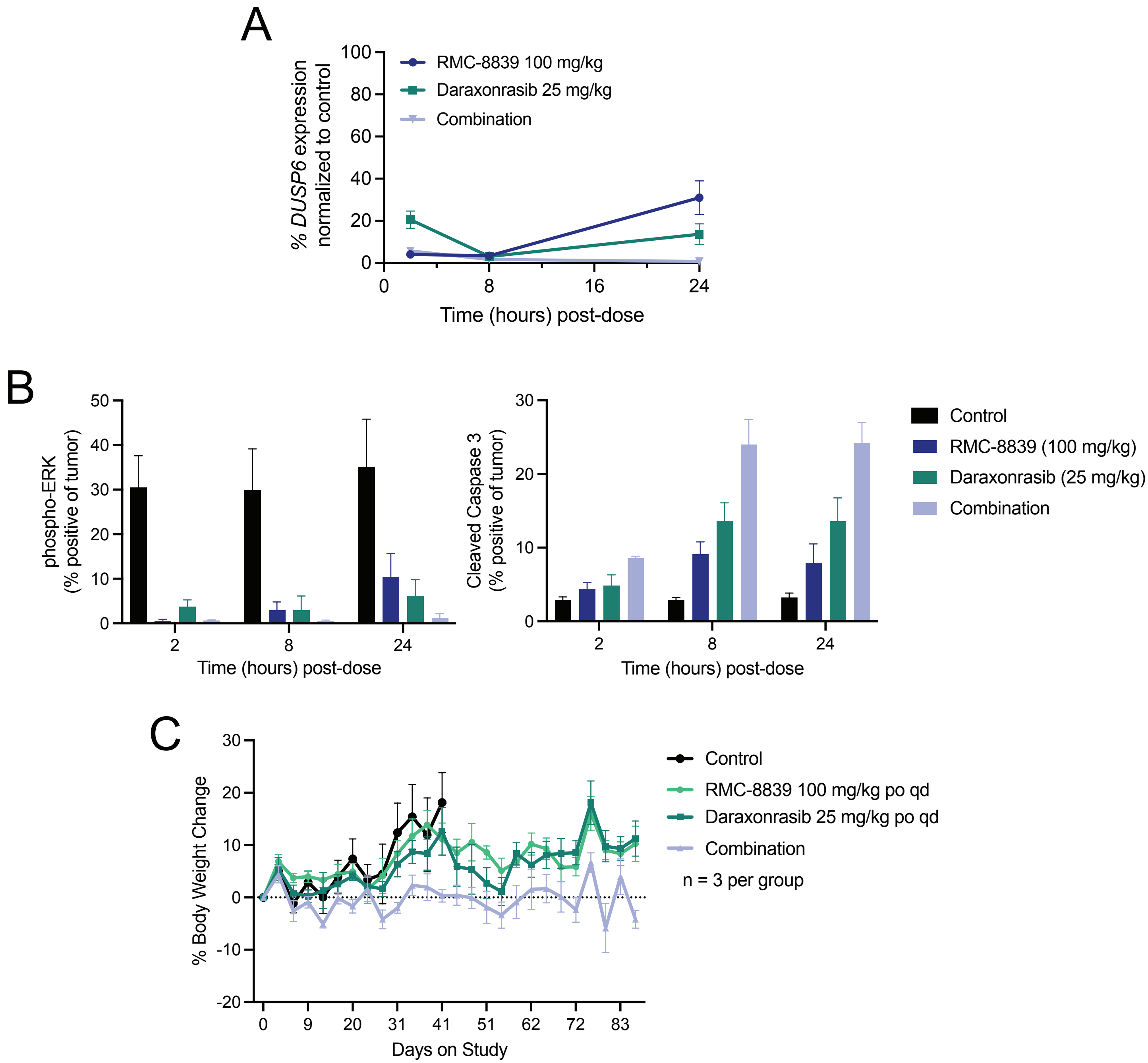

Supplementary Figure S18: Combination of RMC-8839 and daraxonrasib. (A) NCI-H1734 tumors excised at various times after a single dose of RMC-8839, daraxonrasib, or the combination were assessed for expression of DUSP6 mRNA (mean ± SEM, n=3). (B) Quantification of treated NCI-H1734 CDX tumors for phospho-ERK or cleaved caspase 3 (CC3) as assessed by IHC (see Fig. 6C) (C) The body weights of mice treated with vehicle control, RMC-8839 single agent (100 mg/kg po qd), RMC-6236 (25 mg/kg po qd), or the combination therapy were monitored for the 86-day treatment period (n=3, mean ± SEM).
