# Supplementary material for: Selective Inhibition of KRASG13C Reveals an Increased Dependence on Wild-type RAS Isoforms in Codon 13 RAS-Mutant Cancers
Source: Cancer Discov. 2026 Jun 22;16(11):2256–73. doi: 10.1158/2159-8290.CD-25-0886 (PMC13725342; doi:10.1158/2159-8290.CD-25-0886)
Supplement: Supplementary file 10 [file cd-25-0886_figure_s1_suppsf1.pdf]

Figure S1

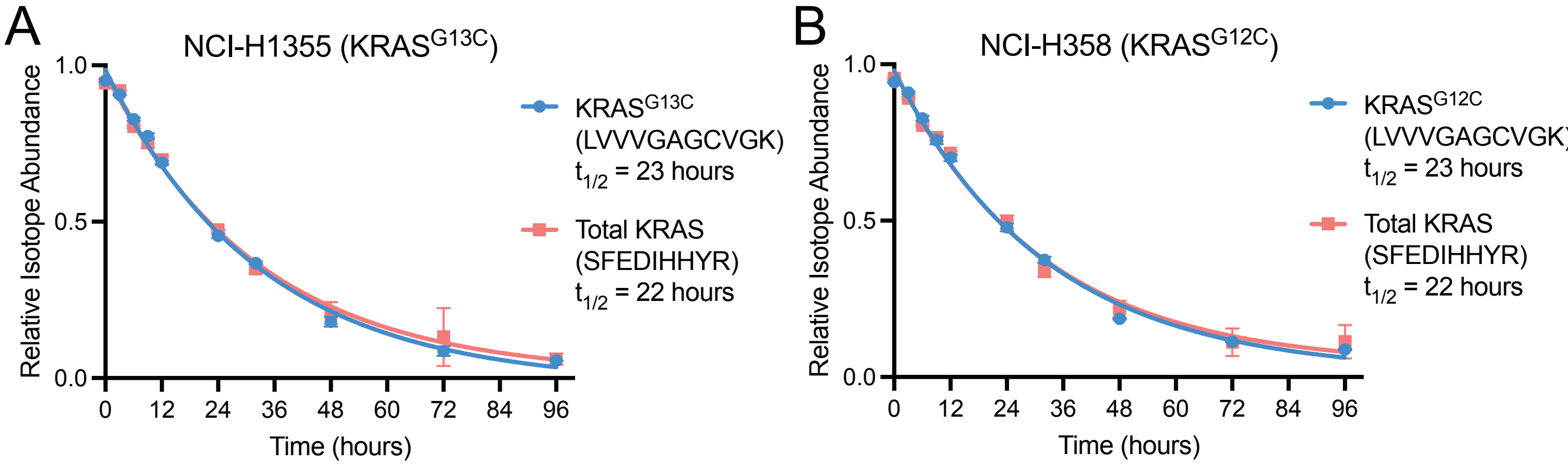

Supplementary Figure S1. Half-Lives of KRASG13C and KRASG12C in cancer cell lines by SILAC. (A) NCI-H1355 or (B) NCI-H358 cells were cultured in Heavy RPMI 1640 for SILAC for more than 5 doublings to ensure complete heavy isotope incorporation. Cells were then re-plated in light RPMI and cells were harvested at after various time points. The relative isotope abundance of the indicated mutant-specific or total KRAS peptide [(Total Area of Heavy Peptide)/(Total Area of Heavy Peptide + Total Area of Light Peptide)] was plotted for each sample.
