# Supplementary material for: Selective Inhibition of KRASG13C Reveals an Increased Dependence on Wild-type RAS Isoforms in Codon 13 RAS-Mutant Cancers
Source: Cancer Discov. 2026 Jun 22;16(11):2256–73. doi: 10.1158/2159-8290.CD-25-0886 (PMC13725342; doi:10.1158/2159-8290.CD-25-0886)
Supplement: Supplementary file 11 [file cd-25-0886_figure_s2_suppsf2.pdf]

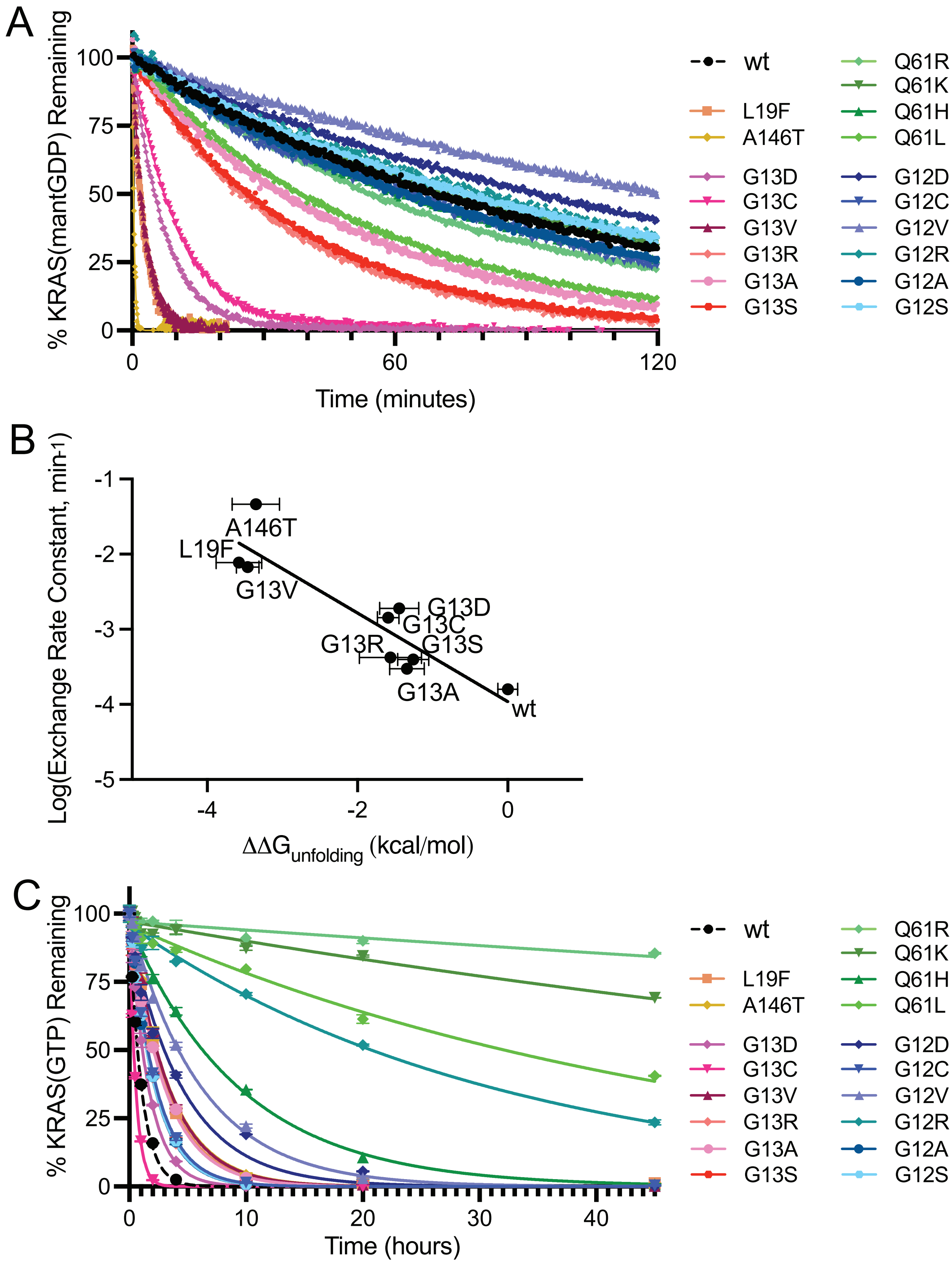

Supplementary Figure S2. Reaction progress curves for the measurement of spontaneous nucleotide exchange and intrinsic nucleotide hydrolysis. (A) The fluorescence of mantGDP-bound KRAS protein was normalized to initial and final values, then fit to a single phase exponential to obtain the spontaneous GDP exchange rate constant shown in Fig. 1F.(B) The log of the GDP exchange rate constants (Fig. 1F) and ΔG of unfolding relative to wild-type (Fig. 1E) for the destabilized RAS mutants were plotted to assess the correlation (r2 = 0.80). (C) GTP-loaded KRAS proteins were incubated, time points were quenched and the percent GTP remaining was plotted then fit to a single phase exponential to obtain the intrinsic GTP hydrolysis rate constant.
