# Supplementary material for: Selective Inhibition of KRASG13C Reveals an Increased Dependence on Wild-type RAS Isoforms in Codon 13 RAS-Mutant Cancers
Source: Cancer Discov. 2026 Jun 22;16(11):2256–73. doi: 10.1158/2159-8290.CD-25-0886 (PMC13725342; doi:10.1158/2159-8290.CD-25-0886)
Supplement: Supplementary file 12 [file cd-25-0886_figure_s3_suppsf3.pdf]

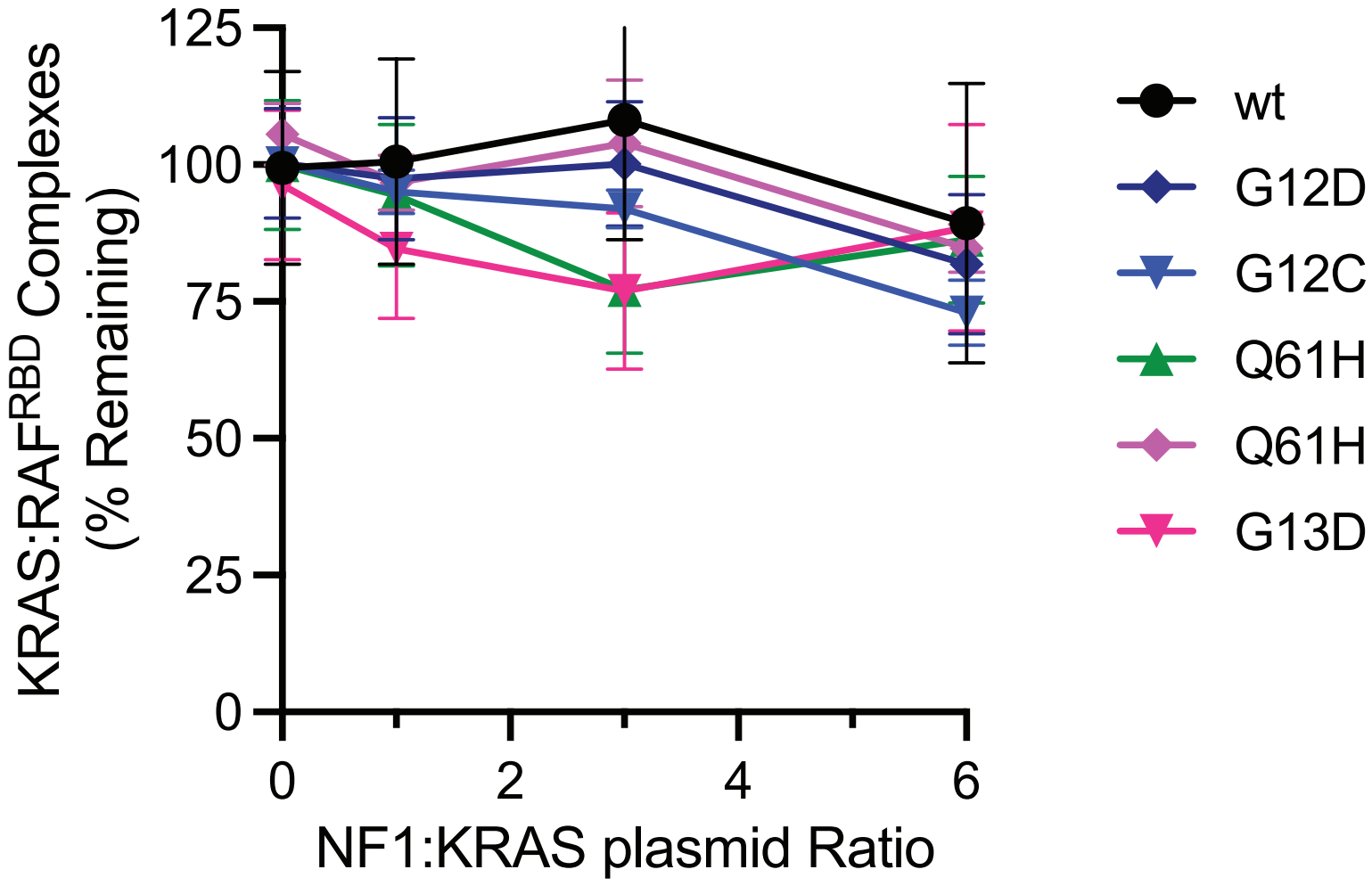

Supplementary Figure S3: Arginine finger mutants of the NF1 catalytic domain abrogates the effect on KRAS:RAF RBD complexes in cells. Cells were co-transfected with KRAS and NF1 plasmid as described in Figure 2C, except that the NF1 plasmid contained the R1276Q mutation previously demonstrated to disrupt catalytic GAP activity.
