# Supplementary material for: Selective Inhibition of KRASG13C Reveals an Increased Dependence on Wild-type RAS Isoforms in Codon 13 RAS-Mutant Cancers
Source: Cancer Discov. 2026 Jun 22;16(11):2256–73. doi: 10.1158/2159-8290.CD-25-0886 (PMC13725342; doi:10.1158/2159-8290.CD-25-0886)
Supplement: Supplementary file 13 [file cd-25-0886_figure_s4_suppsf4.pdf]

Figure S4

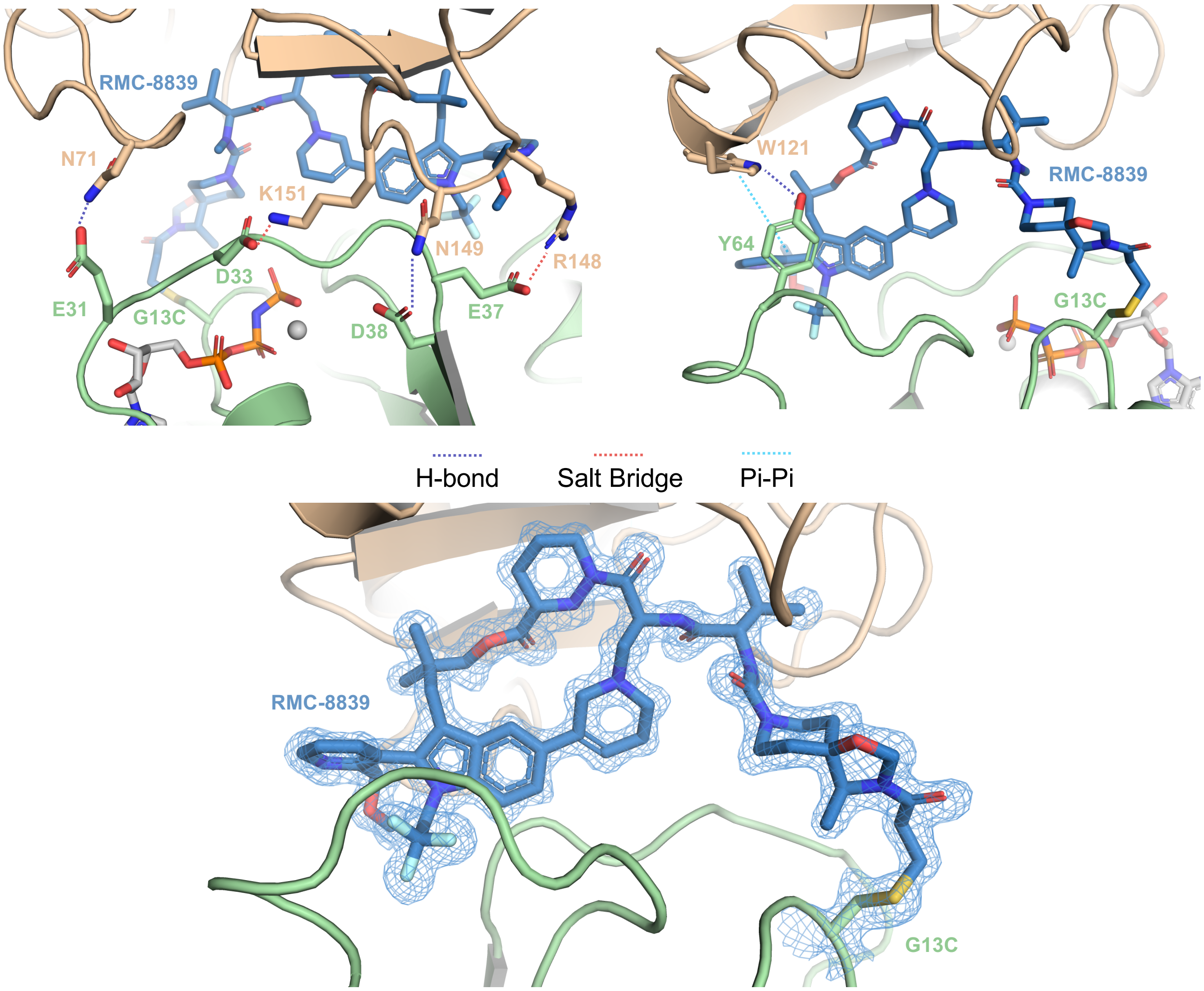

Supplementary Figure S4: Crystal structural characterization of KRAS G13C-CypA tri-complex induced by RMC-8839. KRAS G13C (green), RMC-8839 (blue), and CypA (wheat) tri-complex is shown. Interactions between CypA and RAS switch I (left) and RAS switch II (right) are indicated with dashed lines. The bottom panel shows the unbiased omit map (blue mesh) at 2.5 σ with electron density indicating a covalent bond between RMC-8839 and mutant residue G13C.
