# Supplementary material for: Selective Inhibition of KRASG13C Reveals an Increased Dependence on Wild-type RAS Isoforms in Codon 13 RAS-Mutant Cancers
Source: Cancer Discov. 2026 Jun 22;16(11):2256–73. doi: 10.1158/2159-8290.CD-25-0886 (PMC13725342; doi:10.1158/2159-8290.CD-25-0886)
Supplement: Supplementary file 14 [file cd-25-0886_figure_s5_suppsf5.pdf]

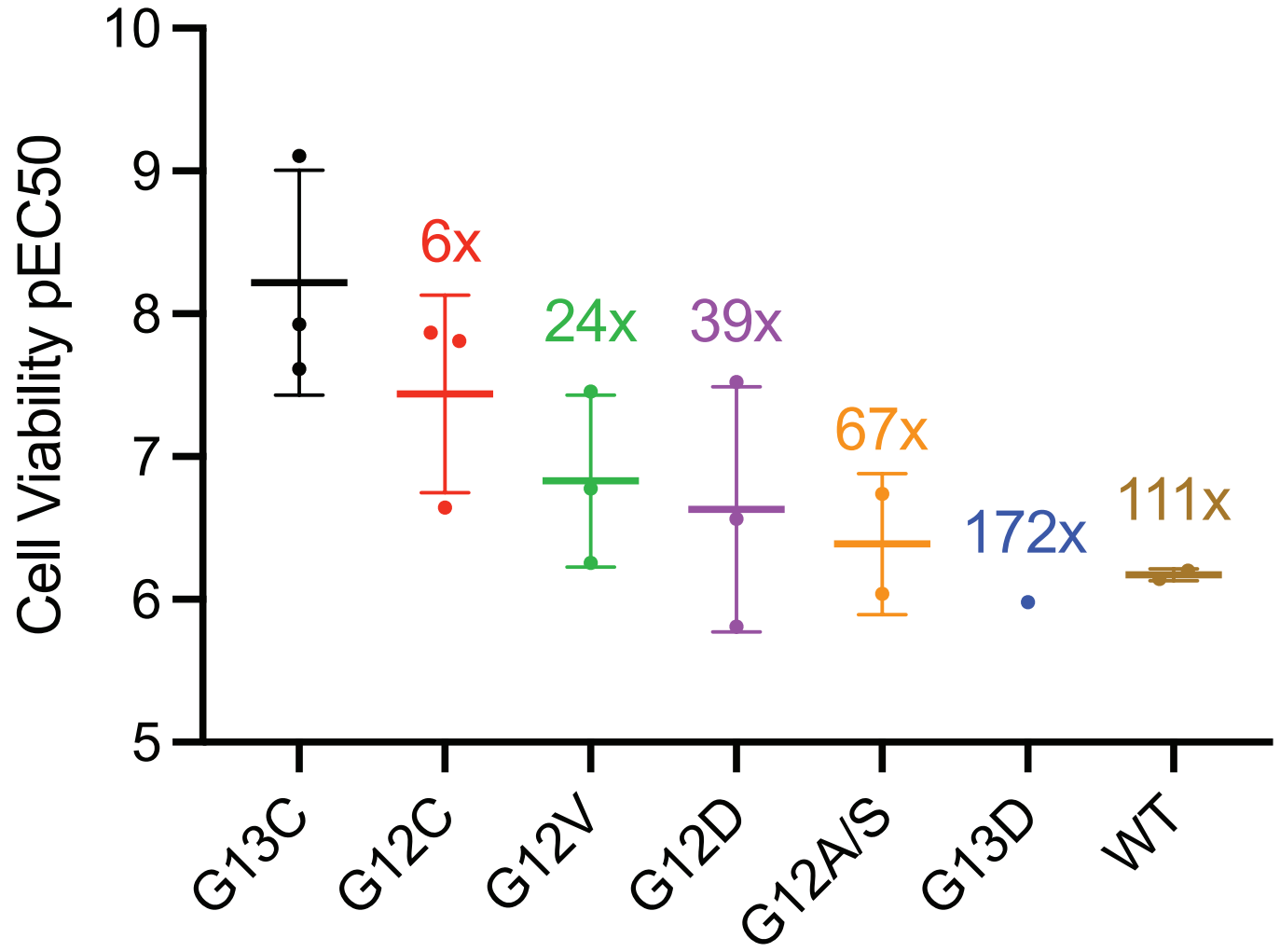


Supplementary Figure S5: RMC-8839 is selective for KRAS G13C over other RAS-mutant NSCLC Cancer Cell Lines. NSCLC cancer cell lines were seeded in 3D spheroid format and treated with RMC-8839 for 5 days. The pEC50 for inhibiting cell viability is plotted for the cell lines containing each KRAS mutation and the mean selectivity for that mutant vs. G13C is indicated above each bar. The cell lines tested were G13C [NCI-H1734, NCI-H1355, MOR], G12C [NCI-H358, LU65, CALU1], G12D [T3M10, A427, SKLU1], G12V [NCI-H441, SW900, H2445], G12A/S [A549, NCI-H2009], G13D [NCI-H1944], wild-type [NCI-H2228, PC9]
