# Supplementary material for: Selective Inhibition of KRASG13C Reveals an Increased Dependence on Wild-type RAS Isoforms in Codon 13 RAS-Mutant Cancers
Source: Cancer Discov. 2026 Jun 22;16(11):2256–73. doi: 10.1158/2159-8290.CD-25-0886 (PMC13725342; doi:10.1158/2159-8290.CD-25-0886)
Supplement: Supplementary file 15 [file cd-25-0886_figure_s6_suppsf6.pdf]

Figure S6

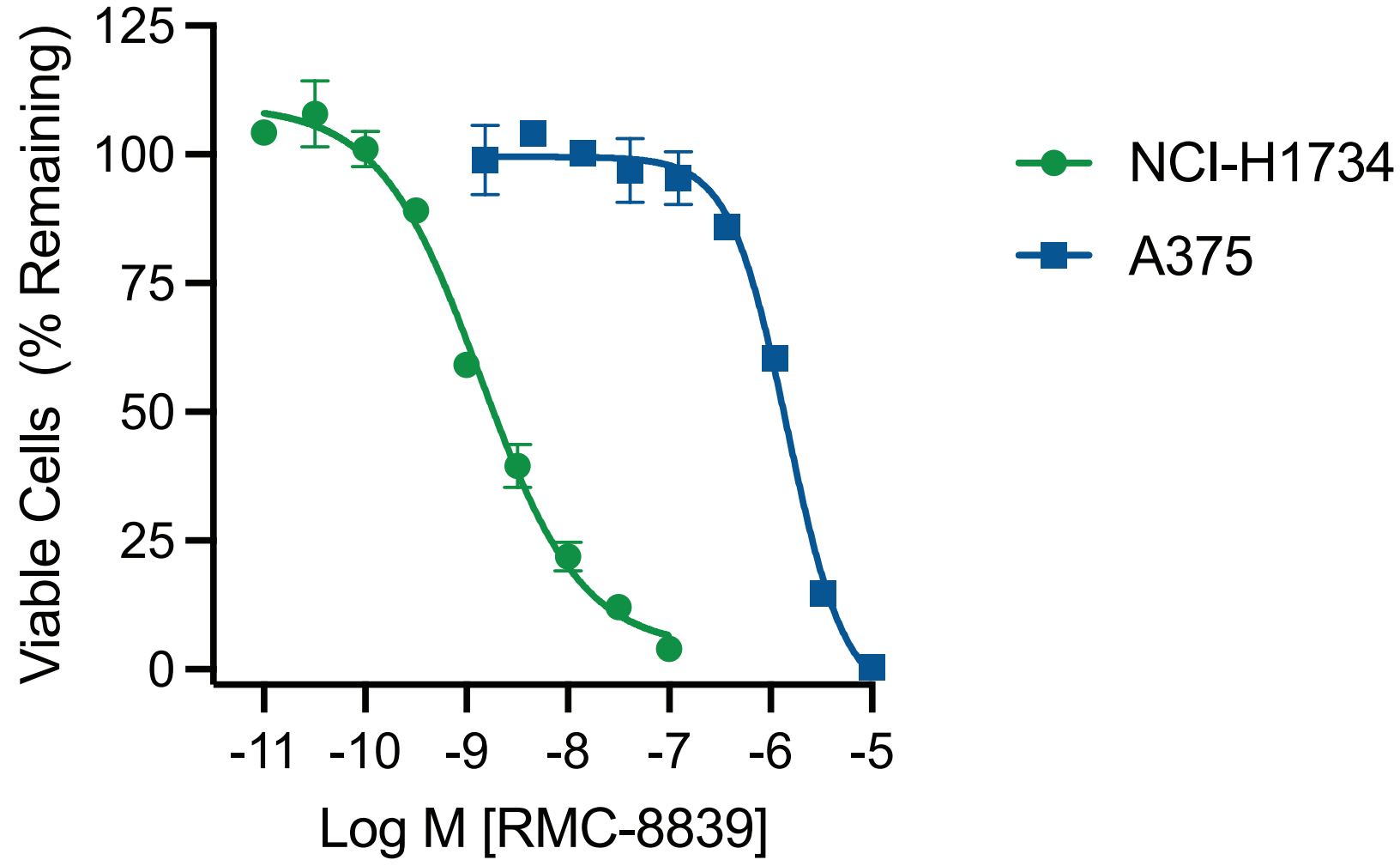

Supplementary Figure S6: RMC-8839 is selective for KRAS G13C over RAS-independent cells. NCI-H1734 (KRAS G13C) or RAS-independent A375 (BRAF V600E) cell lines were treated with RMC-8839 for 5 days and the cell viability was assessed. The potency of RMC-8839 is >1000x greater in NCI-H1734 than the RAS-independent A375 cell line.
