# Supplementary material for: Selective Inhibition of KRASG13C Reveals an Increased Dependence on Wild-type RAS Isoforms in Codon 13 RAS-Mutant Cancers
Source: Cancer Discov. 2026 Jun 22;16(11):2256–73. doi: 10.1158/2159-8290.CD-25-0886 (PMC13725342; doi:10.1158/2159-8290.CD-25-0886)
Supplement: Supplementary file 16 [file cd-25-0886_figure_s7_suppsf7.pdf]

Figure S7

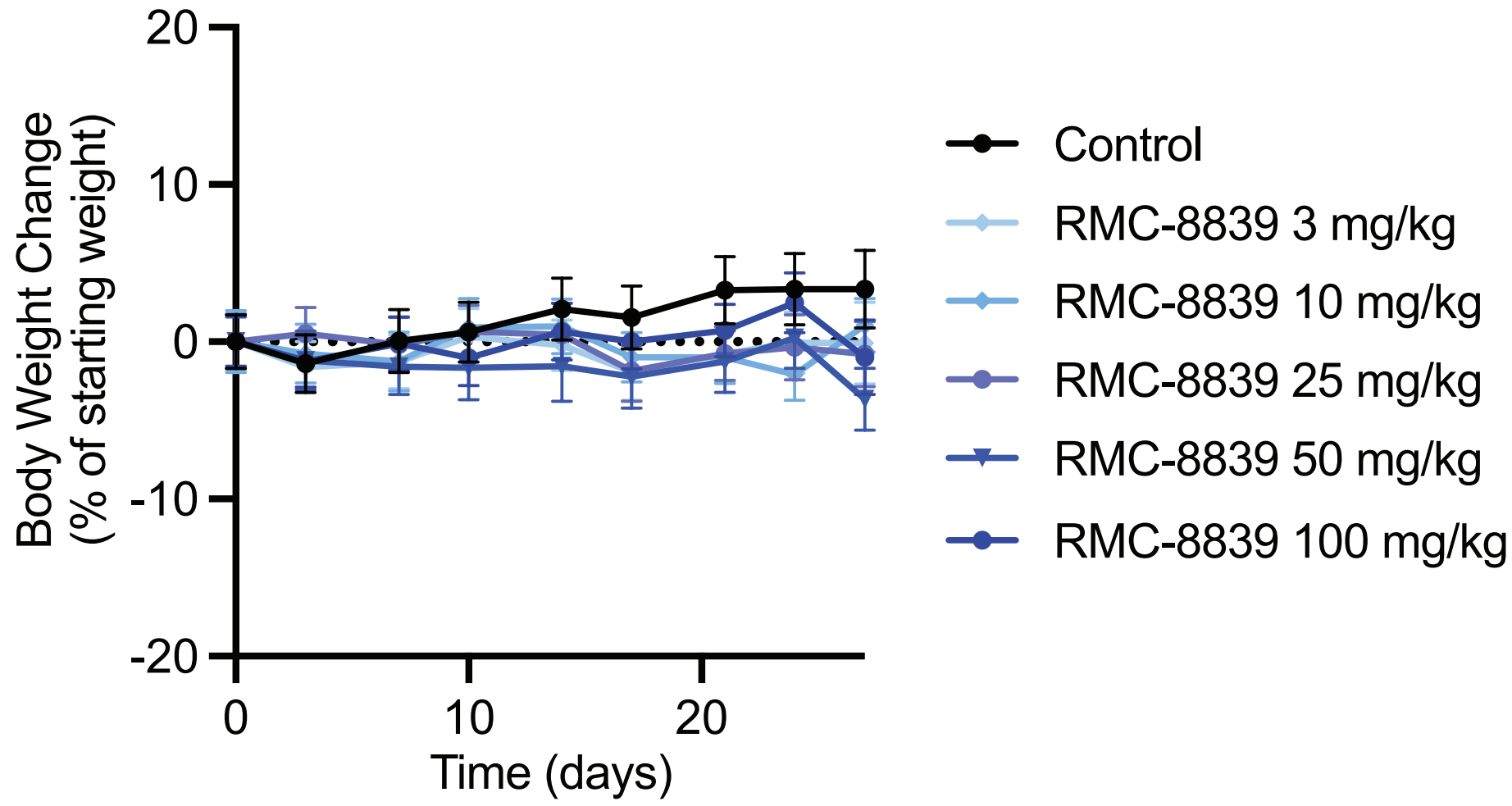

Supplementary Figure S7: RMC-8839 was well-tolerated at daily doses up to 100 mg/kg. Mice bearing NCI-H1734 tumors were treated once daily with the indicated oral dose and the mouse body weights were monitored for 28 days.
