# Supplementary material for: Selective Inhibition of KRASG13C Reveals an Increased Dependence on Wild-type RAS Isoforms in Codon 13 RAS-Mutant Cancers
Source: Cancer Discov. 2026 Jun 22;16(11):2256–73. doi: 10.1158/2159-8290.CD-25-0886 (PMC13725342; doi:10.1158/2159-8290.CD-25-0886)
Supplement: Supplementary file 17 [file cd-25-0886_figure_s8_suppsf8.pdf]

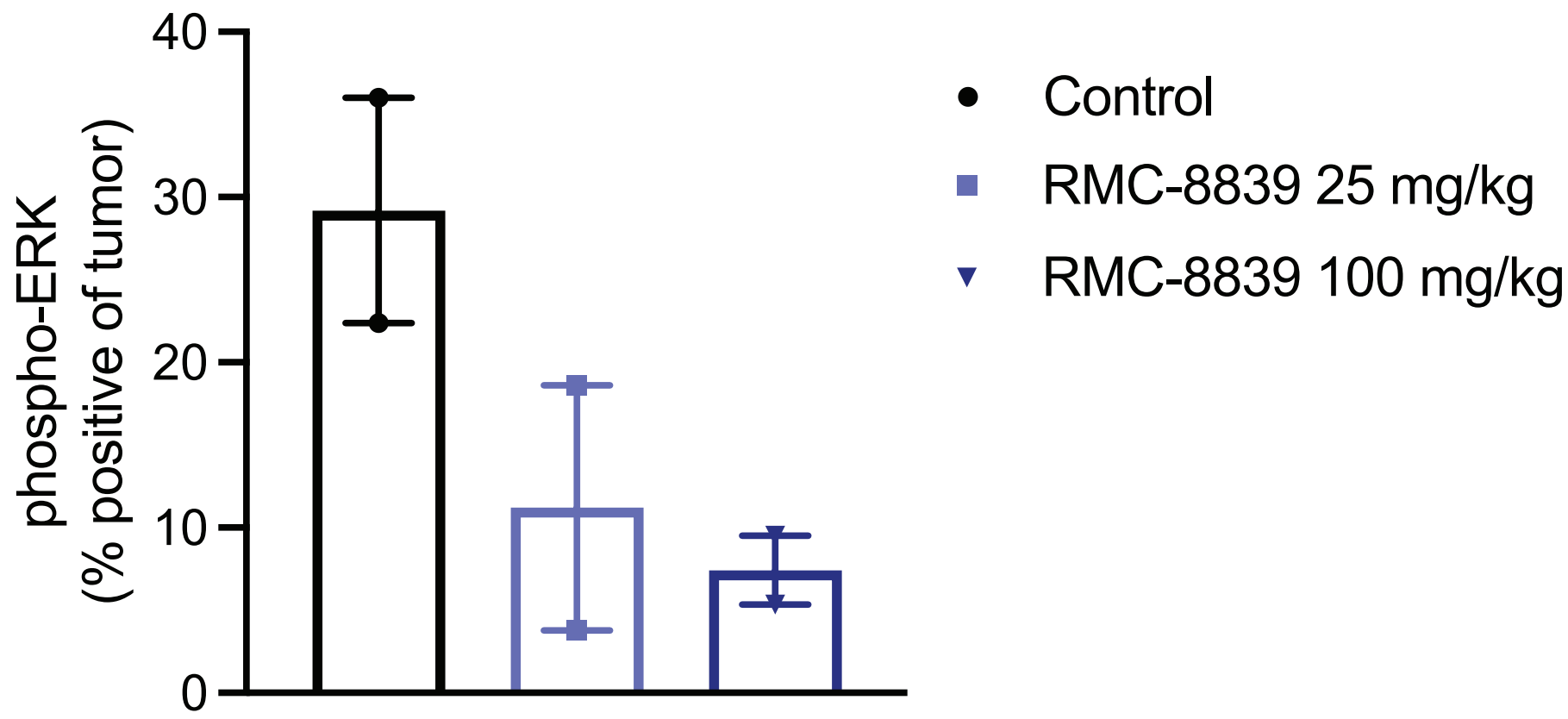

Supplementary Figure S8: Suppression of MAPK Pathway Signaling at End of 28-day Study. (A) Mice bearing NCI-H1734 CDX tumors were treated once daily with the indicated oral doses of RMC-8839 for 28 days. Tumors were collected 8 hours after the final dose and pERK was assessed by IHC.
