# Supplementary material for: Selective Inhibition of KRASG13C Reveals an Increased Dependence on Wild-type RAS Isoforms in Codon 13 RAS-Mutant Cancers
Source: Cancer Discov. 2026 Jun 22;16(11):2256–73. doi: 10.1158/2159-8290.CD-25-0886 (PMC13725342; doi:10.1158/2159-8290.CD-25-0886)
Supplement: Supplementary file 18 [file cd-25-0886_figure_s9_suppsf9.pdf]

Figure S9

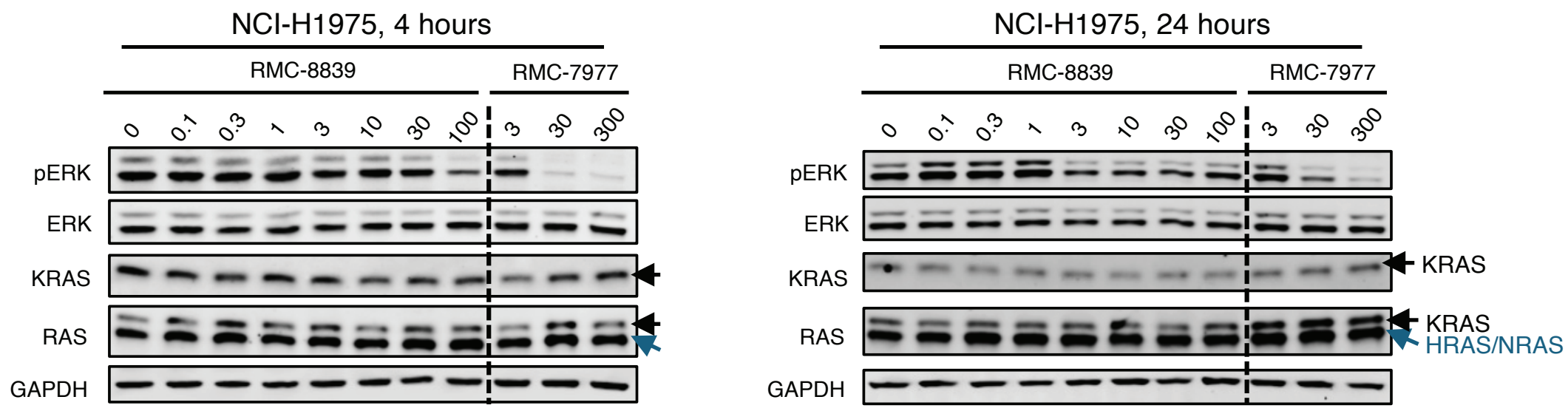

Supplementary Figure S9: RMC-8839 effects on cellular signaling in RAS wild-type cell line. The wild-type RAS cell line NCI-H1975 was treated with the indicated concentration of RMC-8839 or RMC-7977 and lysed after 4 hours (left) or 24 hours (right) of treatment.
