# Supplementary material for: Selective Inhibition of KRASG13C Reveals an Increased Dependence on Wild-type RAS Isoforms in Codon 13 RAS-Mutant Cancers
Source: Cancer Discov. 2026 Jun 22;16(11):2256–73. doi: 10.1158/2159-8290.CD-25-0886 (PMC13725342; doi:10.1158/2159-8290.CD-25-0886)
Supplement: Supplementary file 19 [file cd-25-0886_table_s1_suppst1.docx]

**Supplementary Table S1**

Table S1: Protein crystallography refinement parameters

|  | KRAS^WT^ | KRAS^G13C^ | KRAS^G13C^ | KRAS^G13C^ | KRAS^G13C^ |
| --- | --- | --- | --- | --- | --- |
|  |  |  | CYPA | CYPA | CYPA |
|  |  |  | Cmpd-1 | Cmpd-2 | RMC-8839 |
| **Data collection*** |  |  |  |  |  |
| Space group | P 2_1_ 2_1_ 2_1_ | C 1 2 1 | P 1 2_1_ 1 | P 1 2 1 | P 1 2_1_ 1 |
| Cell dimensions |  |  |  |  |  |
| *a*, *b*, *c* (Å) | 48.9, 63.0, 119.4 | 63.7, 38.5, 69.8 | 48.1, 105.1, 66.1 | 64.8, 48.1, 101.7 | 48.0, 101.7, 66.2 |
| α, β, γ (°) | 90, 90, 90 | 90, 113, 90 | 90, 91, 90 | 90, 93, 90 | 90, 91, 90 |
| Resolution (Å)** | 2.05 (2.13-2.05) | 1.25 (1.28-1.25) | 1.47 (1.49-1.47) | 1.74 (1.77-1.74) | 1.21 (1.22-1.21) |
| *R*_merge_ | 0.187 (1.20) | 0.063 (1.58) | 0.0792 (1.97) | 0.0716 (1.84) | 0.083 (1.15) |
| *I* / σ*I* | 8.3 (2.1) | 7.74 (1.01) | 7.43 (0.63) | 7.69 (0.51) | 6.33 (0.86) |
| Completeness (%) | 100 (100) | 98.50 (98.19) | 98.19 (98.63) | 97.98 (93.11) | 97.98 (93.89) |
| Redundancy | 10.0 (10.3) | 3.7 (3.7) | 3.8 (3.9) | 3.9 (3.6) | 3.8 (3.8) |
|  |  |  |  |  |  |
| **Refinement** |  |  |  |  |  |
| Resolution (Å) | 2.05 | 1.25 | 1.47 | 1.74 | 1.21 |
| No. reflections | 23828 (2567) | 158781 (10320) | 414232 (14144) | 63319 (2717) | 719656 (22905) |
| *R*_work_ / *R*_free_ | 18.0/23.4 | 16.9/35.9 | 19.2/23.8 | 18.3/21.8 | 13.6/17.8 |
| No. atoms |  |  |  |  |  |
| Protein | 2704 | 1391 | 5259 | 5261 | 5374 |
| Ligand/ion | 70 | 37 | 196 | 222 | 223 |
| Water | 260 | 141 | 695 | 394 | 1042 |
| *B*-factors |  |  |  |  |  |
| Protein | 30.65 | 25.96 | 25.47 | 39.81 | 19.19 |
| Ligand/ion | 23.21 | 19.73 | 19.09 | 32.49 | 16.43 |
| Water | 36.97 | 40.25 | 37.85 | 42.41 | 39.64 |
| R.m.s. deviations |  |  |  |  |  |
| Bond lengths (Å) | 0.007 | 0.005 | 0.006 | 0.007 | 0.07 |
| Bond angles (°) | 0.88 | 0.78 | 0.82 | 0.84 | 0.94 |

*Each dataset was obtained from a single crystal.

**Values in parentheses are for highest-resolution shell.
