# Supplementary material for: Selective Inhibition of KRASG13C Reveals an Increased Dependence on Wild-type RAS Isoforms in Codon 13 RAS-Mutant Cancers
Source: Cancer Discov. 2026 Jun 22;16(11):2256–73. doi: 10.1158/2159-8290.CD-25-0886 (PMC13725342; doi:10.1158/2159-8290.CD-25-0886)
Supplement: Supplementary file 20 [file cd-25-0886_table_s2_suppst2.docx]

**Supplementary Table S2**

Table S2: BRAF mutation classification and prevalence in KRAS^G13X^ NSCLC

| **BRAF Mutation** | **BRAF Classification** | **KRAS^G13C^ NSCLC**  **Prevalence** | **KRAS^G13D^ NSCLC**  **Prevalence** |
| --- | --- | --- | --- |
| G466V | Class 3 | 1.20% | 0.10% |
| G464V | Class 2 | 0.80% | N/D |
| G469V | Class 2 | 0.60% | 0.40% |
| N581S | Class 3 | 0.50% | 0.10% |
| G469A | Class 2 | 0.40% | N/D |
| G469R | Class 2 | 0.40% | 0.10% |
| G466A | Class 3 | 0.40% | N/D |
| N581I | Class 3 | 0.30% | 0.10% |
| V471F | Class 2 | 0.20% | N/D |
| G596R | Class 3 | 0.20% | N/D |
| E501K | Class 3 | 0.20% | N/D |
| D594N | Class 3 | 0.20% | 0.20% |
| L485W | Class 2 | 0.20% | N/D |
| D594G | Class 3 | 0.20% | N/D |
| K601N | Class 2 | 0.10% | N/D |
| V600G | Class 1 | 0.10% | N/D |
| E586K | Class 2 | 0.10% | N/D |
| G466S | Class 3 | 0.10% | N/D |
| N581H | Class 3 | 0.10% | N/D |
| N581Y | Class 3 | 0.10% | N/D |
| G466R | Class 3 | 0.10% | 0.10% |
| V600E | Class 1 | 0.10% | 0.30% |
| G596C | Class 3 | 0.10% | 0.10% |
| V600L | Class 1 | 0.10% | N/D |
| L485F | Class 2 | 0.10% | N/D |
| G466E | Class 3 | 0.10% | 0.20% |
| D594A | Class 3 | 0.10% | 0.10% |
| N581T | Class 3 | N/D | 0.20% |
| G596V | Class 2 | N/D | 0.20% |
| Y198H | Unknown | N/D | 0.10% |
| G469E | Class 3 | N/D | 0.10% |
| S363F | Class 3 | N/D | 0.10% |
| E501G | Unknown | N/D | 0.10% |
| D594H | Class 3 | N/D | 0.10% |
| A712T | Class 3 | N/D | 0.10% |

N/D = not detected
