# Supplementary material for: Selective Inhibition of KRASG13C Reveals an Increased Dependence on Wild-type RAS Isoforms in Codon 13 RAS-Mutant Cancers
Source: Cancer Discov. 2026 Jun 22;16(11):2256–73. doi: 10.1158/2159-8290.CD-25-0886 (PMC13725342; doi:10.1158/2159-8290.CD-25-0886)
Supplement: Supplementary file 21 [file cd-25-0886_table_s3_suppst3.docx]

**Supplementary Table S3**

Table S3: BRAF mutation classification and prevalence in KRAS^G13D^ CRC

| **BRAF Mutation** | **BRAF Classification** | **KRAS^G13D^ CRC**  **Prevalence** |
| --- | --- | --- |
| D594G | Class 3 | 0.31% |
| V600E | Class 1 | 0.18% |
| D594N | Class 3 | 0.09% |
| G469R | Class 2 | 0.07% |
| G469A | Class 2 | 0.06% |
| G466V | Class 3 | 0.04% |
| G469V | Class 2 | 0.04% |
| D594A | Class 3 | 0.04% |
| K601E | Class 2 | 0.04% |
| T241M | Class 2 | 0.04% |
| N581S | Class 3 | 0.04% |
| A712T | Class 3 | 0.02% |
| D594H | Class 3 | 0.02% |
| G466E | Class 3 | 0.02% |
| K483R | Class 3 | 0.02% |
| Q262P | Class 2 | 0.02% |
| S607P | Class 1 | 0.02% |
| F247L | Class 2 | 0.02% |
| Q201H | Class 3 | 0.02% |
| V471I | Class 3 | 0.02% |
| F357S | Class 3 | 0.02% |
| L597R | Class 3 | 0.02% |
| R444W | Class 1 | 0.02% |
| G466A | Class 3 | 0.02% |
| G466R | Class 1 | 0.02% |
| K483N | Class 2 | 0.02% |
| L485S | Class 2 | 0.02% |
| Y472C | Class 3 | 0.02% |
| D594E | Class 3 | 0.02% |
| K601N | Class 2 | 0.02% |
| S602Y | Unknown | 0.02% |
| V600G | Class 3 | 0.02% |
| C532Y | Class 3 | 0.02% |
| E501K | Unknown | 0.02% |
| L597Q | Class 3 | 0.02% |
| N581D | Class 3 | 0.02% |
| T599I | Unknown | 0.02% |
| H608R | Unknown | 0.02% |
| N581Y | Class 3 | 0.02% |
