# Supplementary material for: Selective Inhibition of KRASG13C Reveals an Increased Dependence on Wild-type RAS Isoforms in Codon 13 RAS-Mutant Cancers
Source: Cancer Discov. 2026 Jun 22;16(11):2256–73. doi: 10.1158/2159-8290.CD-25-0886 (PMC13725342; doi:10.1158/2159-8290.CD-25-0886)
Supplement: Supplementary file 22 [file cd-25-0886_table_s4_suppst4.docx]

**Supplementary Table S4**

Table S4: RTK Mutations in KRAS^G12X^ and KRAS^G13X^ Tumors

| **Comparison** | **Odds Ratio** | **P value** |
| --- | --- | --- |
| G13X vs. G12X NSCLC | 1.25 | 0.00025 |
| G13D vs. G12D CRC | 1.16 | 0.00014 |

Known or likely functional mutations in ALK, EGFR, ERBB2, ERBB3, ERBB4, FGFR1, FGFR2, FGFR3, FGFR4, FLT3, IGF1R, IRS2, JAK2, KIT, KIT, MET, NTRK1, NTRK2, NTRK3, PDGFRA, PDGFRB, RET, or ROS1.
