# Supplementary material for: Selective Inhibition of KRASG13C Reveals an Increased Dependence on Wild-type RAS Isoforms in Codon 13 RAS-Mutant Cancers
Source: Cancer Discov. 2026 Jun 22;16(11):2256–73. doi: 10.1158/2159-8290.CD-25-0886 (PMC13725342; doi:10.1158/2159-8290.CD-25-0886)
Supplement: Supplementary file 23 [file cd-25-0886_table_s5_suppst5.docx]

**Supplementary Table S5**

Table S5: Co-mutations in KRAS^G13C^-Mutant Cancer Cell Lines

|  |  |  | **VAF** | | | | | |
| --- | --- | --- | --- | --- | --- | --- | --- | --- |
| **gene_symbol** | **residue_change** | **oncogenic** | **NCI-H1355** | **NCI-H1734** | **MOR/CPR** | **TOV-21G** | **KKU-213** | **MOR** |
| **KRAS** | p.G13C | Oncogenic | 0.447 | 0.737 | 1.00 | 0.571 | 0.998 | 1.00 |
| **PIK3CA** | p.H1047Y | Oncogenic |  |  |  | 0.455 |  |  |
| **CTNNB1** | p.S33F | Likely Oncogenic |  |  |  |  |  | 0.525 |
| **CTNNB1** | p.S33L | Likely Oncogenic |  |  | 0.433 |  |  |  |
| **CTNNB1** | p.S33P | Likely Oncogenic |  |  |  |  |  | 0.526 |
| **AMER1** | p.E648Ter | Likely Oncogenic |  |  |  | 0.150 |  |  |
| **ARID1A** | p.Y551LfsTer72 | Likely Oncogenic |  |  |  | 0.455 |  |  |
| **ARID1B** | p.K2100EfsTer20 | Likely Oncogenic |  |  |  | 0.647 |  |  |
| **ARID1B** | p.S508Ter | Likely Oncogenic |  |  |  | 0.182 |  |  |
| **ARID5B** | p.G1064AfsTer43 | Likely Oncogenic |  |  |  | 0.489 |  |  |
| **ASXL1** | p.G645Vfs*58 | Likely Oncogenic |  |  |  |  |  | 0.023 |
| **ASXL1** | p.G645VfsTer58 | Likely Oncogenic |  |  |  | 0.857 |  |  |
| **CBFA2T3** | p.E411Ter | Likely Oncogenic |  |  |  | 0.235 |  |  |
| **CDKN2A** | p.D74P | Likely Oncogenic |  | 0.235 |  |  |  |  |
| **CDKN2B** | p.A23V | Likely Oncogenic |  |  |  | 0.25 |  |  |
| **CIC** | p.P1594Afs*20 | Likely Oncogenic |  |  |  |  |  | 0.035 |
| **ELF3** | p.Q151RfsTer4 | Likely Oncogenic |  |  |  |  | 1.00 |  |
| **EP300** | p.Q1725Ter | Likely Oncogenic |  |  | 0.479 |  |  |  |
| **EP400** | p.A30Gfs*38 | Likely Oncogenic |  |  |  |  |  | 0.088 |
| **EP400** | p.N33Pfs*31 | Likely Oncogenic |  |  |  |  |  | 0.058 |
| **EP400** | p.Q2511Pfs*68 | Likely Oncogenic |  |  |  |  |  | 0.014 |
| **EP400** | p.S37Pfs*27 | Likely Oncogenic |  |  |  |  |  | 0.017 |
| **FANCD2** | p.V379= | Likely Oncogenic |  |  |  |  |  | 0.199 |
| **HLA-C** | p.E70Ter | Likely Oncogenic |  |  |  | 0.214 |  |  |
| **INPPL1** | p.R1156GfsTer46 | Likely Oncogenic |  |  |  | 1.00 |  |  |
| **JAK1** | p.K860NfsTer16 | Likely Oncogenic |  |  |  | 0.427 |  |  |
| **JAK1** | p.N339IfsTer3 | Likely Oncogenic |  |  |  | 0.484 |  |  |
| **JARID2** | p.A396RfsTer18 | Likely Oncogenic |  |  |  | 0.284 |  |  |
| **KEAP1** | p.K551Sfs*44 | Likely Oncogenic |  |  |  |  |  | 1.00 |
| **KEAP1** | p.K551SfsTer44 | Likely Oncogenic |  |  | 1.00 |  |  |  |
| **KEAP1** | p.Q75Ter | Likely Oncogenic | 1.00 |  |  |  |  |  |
| **KMT2B** | p.G1099AfsTer83 | Likely Oncogenic |  |  |  | 0.25 |  |  |
| **KMT2C** | p.L4126Ter | Likely Oncogenic |  |  |  | 0.427 |  |  |
| **KMT2D** | p.K4432NfsTer52 | Likely Oncogenic |  |  |  | 0.52 |  |  |
| **MEN1** | p.W265Ter | Likely Oncogenic |  |  |  | 0.632 |  |  |
| **MIDEAS** | p.T1025QfsTer26 | Likely Oncogenic |  |  |  | 0.440 |  |  |
| **NF1** | p.P678RfsTer10 | Likely Oncogenic |  |  |  | 0.341 |  |  |
| **NSD1** | p.R1031Ter | Likely Oncogenic |  |  |  | 0.490 |  |  |
| **PRKDC** | p.A1294Lfs*4 | Likely Oncogenic |  |  |  |  |  | 0.013 |
| **PRKDC** | p.F1296* | Likely Oncogenic |  |  |  |  |  | 0.013 |
| **PTEN** | p.K267RfsTer9 | Likely Oncogenic |  |  |  | 0.36 |  |  |
| **PTPN13** | p.N2237TfsTer60 | Likely Oncogenic |  |  |  | 0.591 |  |  |
| **PTPRT** | p.Q425Ter | Likely Oncogenic |  |  |  | 0.351 |  |  |
| **RBM10** | p.E748Ter | Likely Oncogenic |  |  |  |  | 1.00 |  |
| **SMARCA4** | p.E419Ter | Likely Oncogenic |  |  |  | 0.136 |  |  |
| **STK11** | p.E130* | Likely Oncogenic |  |  |  |  |  | 1.00 |
| **STK11** | p.E130Ter | Likely Oncogenic |  |  | 0.995 |  |  |  |
| **STK11** | p.K48SfsTer3 | Likely Oncogenic | 1.00 |  |  |  |  |  |
| **STK11** | p.M51IfsTer14 | Likely Oncogenic |  | 1.00 |  |  |  |  |
| **STK11** | p.P281RfsTer6 | Likely Oncogenic |  |  |  | 1.00 |  |  |
| **TCF3** | p.G99Ter | Likely Oncogenic |  |  |  | 0.25 |  |  |
| **TP53** | p.E285K | Likely Oncogenic | 1.00 |  |  |  |  |  |
| **TP53** | p.P152Rfs*18 | Likely Oncogenic |  |  |  |  |  | 1.00 |
| **TP53** | p.P152RfsTer18 | Likely Oncogenic |  |  | 1.00 |  |  |  |
| **TP53** | p.R273L | Likely Oncogenic |  | 1.00 |  |  |  |  |
| **ZFHX3** | p.A3407LfsTer78 | Likely Oncogenic |  |  |  | 0.423 |  |  |
| **ZFP36L2** | p.G107AfsTer80 | Likely Oncogenic |  |  |  | 0.571 |  |  |
| **SMAD4** | p.R361C | Likely Oncogenic |  |  |  |  | 1.00 |  |
